# Passivation, phase, and morphology control of CdS nanocrystals probed using fluorinated aromatic amines and solid-state NMR spectroscopy†

**DOI:** 10.1039/d4na00564c

**Published:** 2024-12-18

**Authors:** Mark A. Buckingham, Robert Crawford, Yi Li, Ran Eitan Abutbul, Bing Han, Kerry Hazledine, Sarah Cartmell, Alex Walton, Alex S. Eggeman, David J. Lewis, Daniel Lee

**Affiliations:** a Department of Materials, The University of Manchester Manchester M13 9PL UK David.lewis-4@manchester.ac.uk; b Department of Chemical Engineering, University of Manchester Manchester M13 9PL UK Daniel.lee@manchester.ac.uk; c Department of Chemistry and the Photon Science Institute, The University of Manchester Oxford Road Manchester M13 9PL UK

## Abstract

Nanocrystals are widely explored for a range of medical, imaging, sensing, and energy conversion applications. CdS nanocrystals have been reported as excellent photocatalysts, with thin film CdS also highly important in photovoltaic devices. To optimise properties of nanocrystals, control over phase, facet, and morphology are vital. Here, CdS nanocrystals were synthesised by the solvothermal decomposition of a Cd xanthate single source precursor. To attempt to control CdS nanocrystal surfaces and morphology, the solvent used in the nanocrystal synthesis was altered from pure trioctylphosphine oxide (TOPO) to a mixed TOPO : fluorinated aromatic amine (3-fluorobenzyl amine (3-FlBzAm) or 3-fluoroaniline (3-FlAn)), where ^19^F provides a sensitive NMR-active surface probe. Powder X-ray diffraction found that the CdS nanocrystals synthesised from TOPO : 3-FlAn solvent mixtures were predominantly cubic whilst the TOPO : 3-FlBzAm synthesised nanocrystals were predominantly hexagonal. Raman spectroscopy identified hexagonal CdS in all samples. Solid-state NMR of ^113^Cd, ^19^F, ^13^C, and ^1^H was employed to investigate the local Cd environments, surface ligands, and ligand interactions. This showed there was a mixture of CdS phases present in all samples and that surfaces were capped with TOPO : fluorinated aromatic amine mixtures, but also that there was a stronger binding affinity of 3-FlBzAm compared with 3-FlAn on the CdS surface, which likely impacts growth mechanisms. This work highlights that fluorinated aromatic amines can be used to probe NC surfaces and also control NC properties through their influence during NC growth.

## Abstract 2

Fluorinated ligands with ^19^F and ^113^Cd solid-state NMR spectroscopy provides a powerful tool for analysis of CdS nanocrystals, evidenced here for aromatic amine capping ligands that influence the resulting morphology.

## Graphical abstract

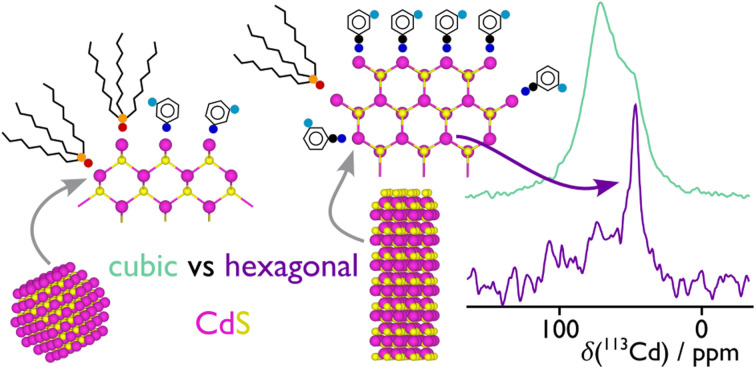

## Introduction

0 Dimension (0D) inorganic nanoparticulate materials are now ubiquitous for medical,^1^ imaging,^2^ sensing,^3^ catalysis,^4–6^ and energy^7^ applications due to their high surface areas, antimicrobial properties, plasmonic fluorescence, and tuneable band gap energies, to name but a few examples. Nanoparticulate materials are therefore extremely attractive in many diverse academic and industrial applications^8^ and are highly tuneable in size and composition to optimise material, catalytic, and optoelectronic properties.

Cadmium sulfide (CdS) is a binary II–VI inorganic semiconductor that is highly promising in solar absorption applications; for example, heterojunction CdS/CdTe photovoltaic devices have demonstrated high efficiencies of up to 20%.^9,10^ CdS has also been extensively explored in photocatalytic reactions,^11–17^ either as a direct photocatalyst for formate photooxidation,^13^ or as a solar absorber with co-catalysts for hydrogen evolution.^11^ CdS has been utilised for these applications as it possesses a band gap energy (*E*_g_) of 2.4 eV, which is commensurate with solar absorption in the visible spectrum.^18,19^

CdS nanoparticles (NPs) have been prepared through a multitude of methods including thermal combustion, microwave assisted, spray pyrolysis, mechanochemical processes, casting techniques, sol gel methods, pulsed laser deposition and colloidal, templated synthesis.^20^ Single source precursor (SSP) routes towards CdS NPs^21,22^ exploit the prefabricated synthesis of discrete inorganic molecular precursors. In this technique, the Cd–S bond is assembled within the precursor itself prior to the decomposition/deposition step, negating any side reactions and pre-reactions in the feed.^23^ Containing potentially toxic metals in a non-volatile, non-pyrophoric, and air-stable precursor is also beneficial from a safety perspective.^23^ Utilising CdS SSPs is also desirable for green chemistry, as this method can reduce the use of highly toxic cadmium salts in techniques such as chemical bath deposition towards the production of CdS thin films.^19^

CdS forms two main phases, hexagonal wurtzite, and cubic sphalerite (also known as zinc blende). In CdS, it has been experimentally demonstrated that under ambient temperature and pressure, cubic sphalerite is more stable than hexagonal wurtzite,^24,25^ with both thin film^25^ and nanoparticulate^24^ CdS forming exclusively wurtzite structured materials at higher temperatures. Theoretical calculations have also shown that sphalerite is a metastable phase and wurtzite is the thermodynamically favourable phase.^26,27^ Tuning the synthesis of cubic and hexagonal phases of CdS NCs has been previously achieved by altering the size of synthesised crystals,^28^ SSP decomposition temperature,^29^ reaction pH,^30^ using cation exchange reactions,^31^ and through altering solvent combinations.^32^ Pure oleylamine and oleylamine mixed with octadecene synthetic regimes were found to form cubic CdS, whereas oleylamine mixed with dodecanethiol formed hexagonal CdS.^32^ Trioctylphosphine oxide (TOPO) produced CdS NCs that are typically hexagonal, rather than cubic,^22,33–35^ and the presence of pyridine and bipyridine complexed to Cd tetrahydroquinoline carbodithioate precursors decomposed in a solvothermal reaction in diethylenetriamine found that pyridine affects the axial growth of hexagonal CdS nanorods.^36^ Phase pure CdS NCs are not always desirable; polymorph mixtures of sphalerite and wurtzite have been reported to improve photocatalytic properties.^37^ Thus, stabilising and achieving control over the crystalline phase formed in reactions to produce NCs is desired and judicious selection of organic solvents for SSP decomposition clearly plays a key structure-directing role in NC synthesis.

The surface of CdS (and other) NPs is generally covered by ligand molecules, which act to passivate the surface,^38,39^ and can also be used to control size, shape, morphology, optoelectronic properties, and surface reactivity.^40^ The particular ligand will depend on the synthesis route and also the desired application, as post-synthesis ligand-exchange is also routinely performed.^41^ Although much work has been undertaken to investigate surface-ligand effects in NPs,^40^ there is still no clear picture of how ligand structure relates to function, which would enable rational design of NPs for specific applications. Very recently, Cao *et. al.* studied the surface features and ligation in alkylamine-capped CdSe NPs and showed that a variety of complex ligand coordination states are possible/present.^42^ Nevertheless, this work studied post-synthetic modification of the NPs with the amine ligands and as such did not address the role ligands can play in NP growth. Furthermore, Chen *et. al.* analysed CdSe nanoplatelets and nanospheroids and determined that they were predominantly terminated with {100} facets and long chain aliphatic ligands, where these ligands were added during the synthesis and likely help control the final morphology.^43^ Long chain aliphatic ligands have been shown to promote the formation of zinc blende CdSe NPs, whereas phosphonate ligands promoted growth of a wurtzite structure;^44^ long chain aliphatic amines only played a secondary role in structure direction in that instance.

Since the decomposition of SSPs to NCs is usually performed in a suitable solvent or solvent mixture where the resulting surface coating of ligands will then stem from the solvent(s), much work has been undertaken to control NC properties using various solvents.^45^ The majority of studies have focussed on long chain alkylamines,^46^ as well as phosphonates, phosphines, phosphine oxides, and thiols^40^ as solvents and capping ligands for Cd-chalcogen NCs. The alkyl chain length of alkylamines can be used to modify the stability of the surface ligands owing to increased ligand–ligand interactions with longer chains, even though amines are generally considered as weak ligands and are in constant exchange with the surface when the NCs are in colloidal solutions/suspension.^46^ For metal oxide NCs, in this case ZnO, it has recently been shown that aromatic groups help stabilise surface ligands through π–π interactions.^47,48^ Moreover, aromatic ligands have been shown to improve the optoelectronic properties of semiconducting NCs owing to their conductivity.^49–51^ Therefore, it is somewhat surprising that ligands employing aromatic groups have rarely been explored in these contexts for Cd-chalcogen NCs.^52^

To better understand how ligand structure relates to NP morphology and surface passivation, atomic-scale characterisation techniques can be employed. Analytical tools such as powder X-ray diffraction (pXRD) and electron microscopy (EM) offer detailed insight into the core structure of NPs, whereas Fourier-transform infrared (FT-IR), Raman, X-ray photoelectron (XPS), and nuclear magnetic resonance (NMR) spectroscopies also allow probing the nature of the surface. Solid-state NMR spectroscopy has been used to study both the surface and bulk of CdS NPs with ^1^H, ^13^C, and ^113^Cd NMR. However, most of this work has been dedicated to characterising the surface and core structure of the inorganic component and has not addressed the key role that ligands play in CdS NP growth and stabilisation. It is therefore of great interest to investigate the effect of ligands on the synthesis of NCs, where the ligand can be probed with atomic-level precision.

Here, we report the synthesis of CdS NPs from an air-stable, single source, inorganic molecular precursor and tune their resulting morphology using a contrasting combination of small aromatic amine-based ligands and a ‘typical’ solvothermal ligand (TOPO). Fluorinated aromatic amines were used as ligands, which are added at the synthesis-stage, to both act as co-capping agents with TOPO, and as an NMR probe to investigate ligand binding affinity and inter-molecular interactions of the capping ligands; ^19^F has very favourable properties for NMR spectroscopy, including high receptivity (∼0.8 relative to ^1^H and ∼4600 relative to ^13^C) and fluorine is rare in natural systems meaning that detected signals are specific to the exogenous fluorinated species. ^19^F solid-state NMR spectroscopy of the fluorinated aromatic ligands provides insight into ligand mixing (with TOPO) at the surface of the NCs as well as a tool to measure distances between aromatic ligands. Depending on the relative ratio of TOPO : fluorinated aromatic amine, and the choice of these amines (3-fluoroaniline or 3-fluorobenzylamine), pXRD and EM show that the size and shape of the CdS NPs can be controlled and pXRD and ^113^Cd NMR show that the prevalence of a particular phase (cubic or hexagonal) can also be manipulated.

## Experimental

### Chemicals

The following chemicals were used without further purification, unless specified: cadmium nitrate tetrahydrate (98%, Sigma-Aldrich), potassium ethylxanthogenate (potassium ethyl xanthate, 96%, Sigma-Aldrich), 3-methyl pyridine (Fluorochem), tetrahydrofuran (THF, ≥99.9%, Sigma-Aldrich), trioctylphosphine oxide (TOPO, 90%, Sigma-Aldrich), 3-fluoroaniline (98+%, Alfa Aesar), 3-fluorobenzylamine (95%, Fluorochem).

### Instrumentation

Solution NMR spectroscopy of the precursor was conducted on a Bruker 400 MHz spectrometer. Elemental analysis (EA) was performed on a ThermoScientific Flash 2000 Organic Elemental Analyzer for CHN and S analyses. Thermogravimetric analysis (TGA) was performed on a Mettler Toledo STARe system under an N_2_ atmosphere and a heat ramp rate of 10 °C min^−1^. Powder X-ray diffraction was measured on a Bruker D8 Discover GIXRD Autochanger with a Cu Kα X-ray source with a wavelength of 1.541874 Å. Raman spectroscopy was performed on a Horiba LabRAM instrument using a 488 nm wavelength laser at 50× magnification. X-ray photoelectron spectroscopy (XPS) was performed using an ESCA2SR spectrometer (ScientaOmicron GmbH) using monochromated Al Kα radiation (1486.6 eV, 20 mA emission at 300 W, 1 mm spot size) with a base pressure of 1 × 10^−9^ mbar. Charge neutralisation was achieved using a low energy electron flood source (FS40A, PreVac). Binding energy scale calibration was performed using C–C in the C 1s photoelectron peak at 284.8 eV. Analysis and curve fitting was performed using Pseudo-Voigt peaks using CasaXPS. Optical measurements were recorded on a Shimadzu UV-1800 in the wavelength range of 1100–300 nm. TEM samples were prepared by drop-casting a suspension of CdS NCs in hexane onto a holey carbon Cu grid. Bright-field (BF) scanning TEM (STEM) images were taken using a Talos 200A field emission electron gun microscope operating at 200 kV. Energy dispersive X-ray spectroscopy (EDS) hyperspectral data were obtained with a Super-X G2 four-segment SDD detector with a probe semi-convergence angle of 10.5 mrad and beam current of approximately 6.54 nA.

### Synthesis of Cd(Ethyl xanthate)_2_(3-methyl pyridine)_2_

Synthesis of [Cd(Xan)_2_(3-mpy)_2_] was adapted from a previous literature procedure.^18,19^ Cadmium nitrate (*ca.* 1 g, 3 mmol) and ethyl xanthate (*ca.* 1 g, 6.2 mmol) were combined in a round bottom flask containing a stirrer bar and placed under an Ar atmosphere. THF (50 mL) was added followed immediately by 3-methyl pyridine (*ca.* 600 μL, 6.2 mmol), and the solution was stirred overnight (typically *ca.* 18 h). The formed precipitate (KNO_3_) was filtered and washed with THF. The solvent in the filtrate was then removed under vacuum and the resulting solid residue was recrystallised in acetone. The successful synthesis of this complex here was confirmed by ^1^H NMR.^19^ Analysis of [Cd(Xan)_2_(3-mpy)_2_]: elemental analysis found (calculated for CdC_18_H_24_N_2_O_2_S_4_ (in %)); C: 40.1 (40.0), H: 4.6 (4.5), N: 5.0, (5.2), S: 23.8, (23.7).

### Synthesis of colloidal CdS nanoparticles

Synthesis of CdS nanocrystals was carried out using a modification of a literature procedure.^22^ TOPO-only synthesis: under an N_2_ atmosphere, TOPO (10 g, 25 mmol) was degassed for 30 min and heated to 160 °C. Simultaneously and separately, 1 g (1.8 mmol) of [Cd(EtXan)_2_(3-mpy)_2_] was dissolved in 5 g (12.5 mmol) of TOPO under an N_2_ atmosphere and heated to 60 °C to fully dissolve the precursor. The Cd precursor solution was then rapidly injected into the hot TOPO, with a further temperature raise to 280 °C for 1 h, yielding an orange colour in the solution. After 1 h, the mixture was allowed to cool below 100 °C and quenched with an excess of methanol, removing any solidified TOPO. Separation of the CdS nanocrystals and removal of excess (unbound) ligand solvent was achieved with the use of a centrifuge at 8*g* for 5 min, which saw the solid nanocrystals separated from methanol and TOPO liquid phase. A further wash was undertaken using toluene (4 mL) to dissolve the solid CdS nanocrystals, to this further methanol (40 mL) was added and again centrifuged at 8*g* for 5 min. The collected orange solid was dried overnight under vacuum. For the dual-capping agent nanocrystal synthesis, the procedure was the same with the exception that the fluorinated ligand replaced the TOPO used to dissolve the CdS precursor, always making up 15 g total. For the 2 : 7 molar ratio TOPO : 3-FlAn, this was a 1 : 1 ratio by mass and was made up to this equivalence in both TOPO solutions.

### Solid-state NMR

Solid-state NMR spectra were recorded using a Bruker 9.4 T (400 MHz ^1^H Larmor frequency) AVANCE III spectrometer equipped with a 4 mm HFX MAS probe. Experiments were acquired at ambient temperature using various MAS rates. Samples were packed into 4 mm o.d. zirconia rotors under ambient conditions, and sealed with a Kel-F rotor cap. The ^1^H (π/2)- and π-pulse durations were 2.5 and 5.0 μs, respectively, the ^13^C (π/2)- and π-pulse durations were 5.0 and 10.0 μs, respectively, the ^19^F (π/2)- and π-pulse durations were 4.8 and 9.6 μs, respectively, and the ^113^Cd (π/2)- and π-pulse durations were 5.0 and 10.0 μs, respectively. The ^113^Cd MAS NMR spectra were recorded using an echo sequence, 
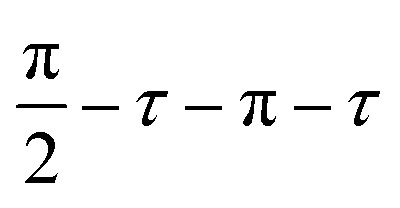
, where the echo time (*τ*) was set to one rotor period, using a repetition delay of 50 s for 1212 to 5088 co-added transients. ^1^H MAS NMR spectra were recorded in the same manner as for ^113^Cd, but the repetition delay was 0.3 s for the TOPO and TOPO/3-FlAn CdS NCs and 0.1 s for the TOPO/3-FlBzAm CdS NCs, and 512 transients were co-added for each. ^13^C MAS NMR spectra were recorded using {^1^H–}^13^C cross-polarization (CP) with an echo (*τ*–π–*τ*) of total duration of two rotor periods. ^13^C spin-locking was applied for 2 ms at ∼50 kHz, with corresponding ramped (70–100%) ^1^H spin-locking at 60–80 kHz (depending on MAS frequency) for CPMAS experiments. 100 kHz SPINAL-64 ^53^ heteronuclear ^1^H decoupling was used throughout signal acquisition. 3968 to 6208 transients were co-added, with repetition delays of 2.7 s. ^19^F MAS NMR spectra were recorded using {^1^H–}^19^F cross-polarization (CP) with an echo (*τ*–π–*τ*) of total duration of two rotor periods. ^19^F spin-locking was applied for 1.25 ms at ∼52 kHz, with corresponding ramped (70–100%) ^1^H spin-locking at 60–80 kHz (depending on MAS frequency) for CPMAS experiments. No RF heteronuclear ^1^H decoupling was used, and 2048 to 8192 transients were co-added using the same repetition delays as for the ^1^H MAS NMR experiments. The ^1^H–^19^F 2D heteronuclear dipolar correlation spectra were recorded using the same parameters as for the corresponding {^1^H–}^19^F CPMAS NMR experiments but with a CP mixing time of 1 ms, and with 512 or 1024 co-added transients for each of 32 complex STATES-TPPI increments with an indirect spectral width to match the MAS frequency. The ^19^F 1D homonuclear double-quantum-filtered (DQF) dipolar correlation NMR spectra were recorded using the SPC-5 mixing sequence^54^ with various mixing times, using a MAS frequency of 12 kHz. No RF ^1^H decoupling was employed at any time during the acquisition of these spectra, and the experiments started with a {^1^H–}^19^F CP step. 32 768 transients were co-added for each mixing time, with the same repetition delays as for the {^1^H–}^19^F CP experiments. Spectral simulations were performed in the solid line-shape analysis (SOLA) module v2.2.4 in Bruker TopSpin v4.0.9. Solid-state NMR spectra were referenced to TMS (^1^H and ^13^C, indirectly *via* adamantane), CCl_3_F (^19^F, indirectly *via* PTFE) and Me_2_Cd (^113^Cd, indirectly *via* Cd(NO_3_)_2_(H_2_O)_4_).

## Results and discussion

### Synthesis and structural characterisation of CdS nanocrystals

**Fig. 1:**
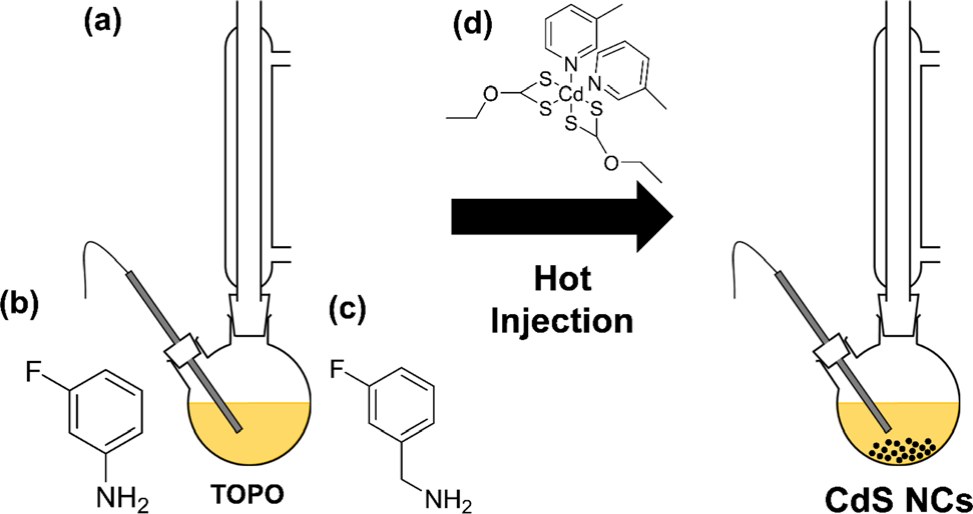
(a) Reaction scheme of the hot injection method to form colloidal CdS nanocrystals. Also shown are the chemical structures of (b) 3-FlAn, (c) 3-FlBzAm and (d) [Cd(Xan)_2_(3-mpy)_2_].

[Cd(ethyl xanthate)_2_(3-methylpyridine)_2_] (hereafter denoted as [Cd(Xan)_2_(3mpy)_2_], Fig. 1(d)) was synthesised following a previously reported procedure.^18,19^ Colloidal CdS nanocrystals (NCs) were synthesised by solvothermally decomposing this precursor in a solution of trioctylphosphine oxide (TOPO) in the presence or absence in various ratios of 3-fluoroaniline (3-FlAn, Fig. 1(b)) or 3-fluorobenzylamine (3-FlBzAm, Fig. 1(c)), as in Table 1 (schematic shown in Fig. 1(a)). Six nanocrystal systems were synthesised based on various molar ratios of TOPO : fluorinated aromatic ligand: (1) TOPO only, (2) 3 : 2 and 2 : 7 TOPO : 3-FlAn and (3) 3 : 2, 3 : 1, and 16 : 1 of TOPO : 3-FlBzAm. This enabled a two-fold investigation: first, to assess the use of mono-fluorinated aromatic ligand as an NMR-active probe on the surface of the NCs and second, to investigate the difference between the benzylamine and aniline functions in terms of aromatic capping agents on CdS NCs and their influence on the resulting material's growth.

**Table 1:** The total mass of solvent (TOPO + either 3-FlAn or 3-FlBzAm) was 15 g

Amount of material/mmol				Ratio TOPO : L (mass)	Ratio TOPO : L (mol)
[Cd(Xan)_2_(mpy)_2_]	TOPO	3-FlAn	3-FlBzAm		
1.85	38.80	—	—	—	—
1.85	32.33	22.50	—	5 : 1	3 : 2
1.85	19.40	67.49	—	1 : 1	2 : 7
1.85	38.02	—	2.40	64 : 1	16 : 1
1.85	35.18	—	11.19	10 : 1	3 : 1
1.85	32.33	—	19.98	5 : 1	3 : 2

**Fig. 2:**
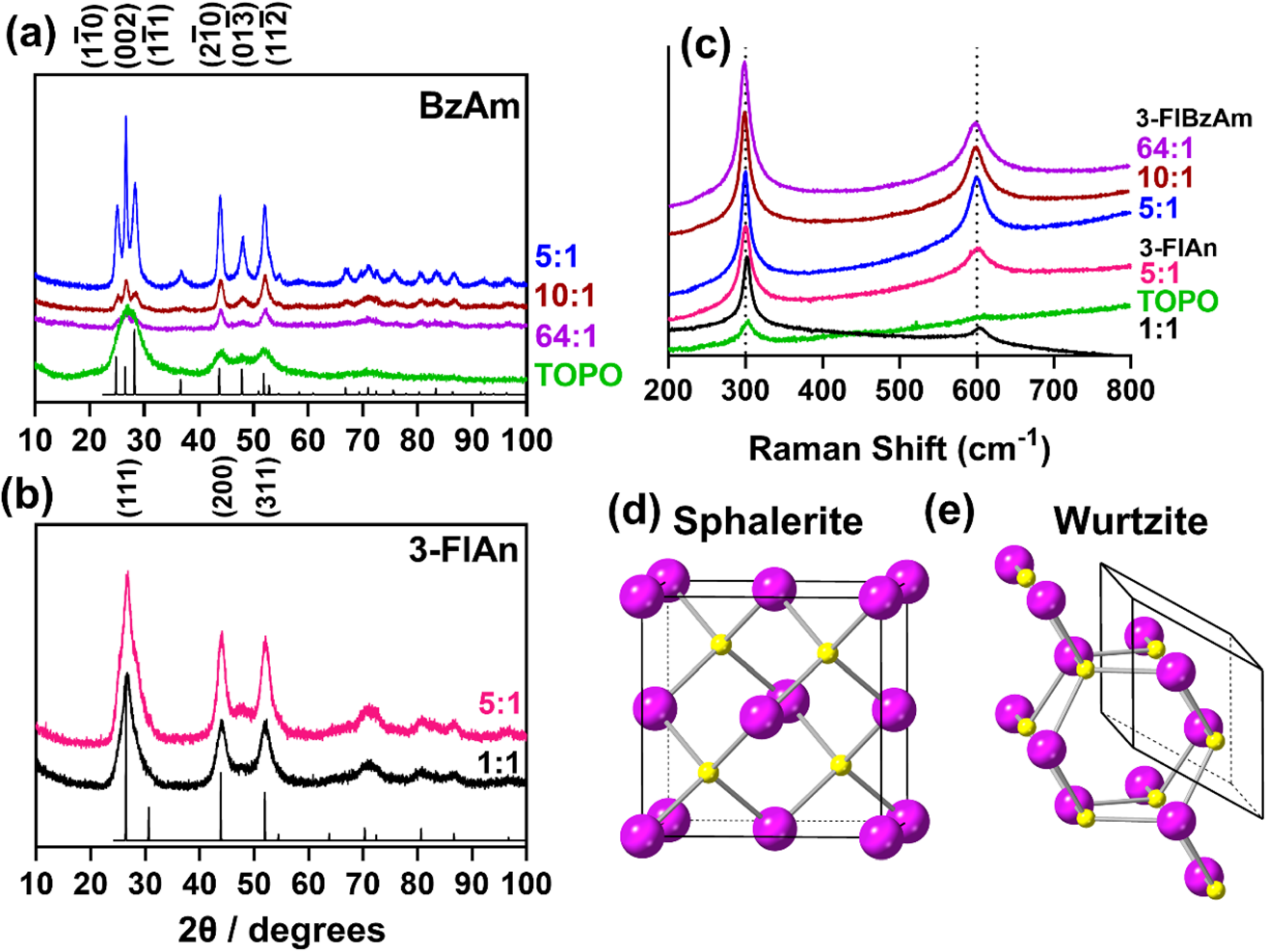
(a and b) pXRD patterns of all investigated CdS nanocrystals with model patterns for (a) hexagonal wurtzite (ICSD: 154186, *P*63*mc*, *a* = 4.1365(3), *b* = 4.1365(3), *c* = 6.7160(4), *α*, *β* = 90°, *γ* = 120°)^55^ and (b) cubic sphalerite (ICSD: 81925, *F*4̄3*m*, *a* = 8304(9), *b* = 5.8304(9), *c* = 5.8304(9), *α*, *β*, *γ* = 90°).^56^ (c) Raman spectra recorded for all synthesised CdS nanocrystals and the unit cells of (d) Sphalerite and (e) Wurtzite with Cd atoms in purple and S atoms in yellow. Ratio of TOPO : fluorinated amine in (a–c) are by mass, as in Table 1.

Powder X-ray diffraction (pXRD, Fig. 2(a and b)) was used to assess the crystalline products of the synthesised nanocrystals. Accurately indexing nanoparticle CdS is challenging due to the Scherrer broadening effect^57^ of small crystallites overlapping the peaks of the two main phases of hexagonal wurtzite CdS (ICSD: 154186) and cubic sphalerite (also known as zinc blende) CdS (ICSD: 81925). The pXRD pattern of the TOPO only CdS system appeared to be consistent with both sphalerite and wurtzite, likely because the NCs are extremely small (and therefore give broad patterns). The pXRD patterns with mixed TOPO :  fluorinated amine systems were easier to index. The pXRD patterns of the three 3-FlBzAm synthesised NCs were found to be wurtzite, with the (11̄0), (002) and (011̄) reflections visible, whilst the 3-FlAn-based NCs were both more consistent with sphalerite, with the absence of any large (013̄) reflection that would be present in wurtzite. In our synthesised nanocrystals, the TOPO mixed with 3-fluoroaniline system is found to stabilise the metastable cubic phase of CdS, but by changing the solvent regime to include 3-fluorobenzyl amine, the thermodynamically favourable wurtzite phase is obtained.

With difficulty determining the exact synthesised phase from pXRD due to the Scherrer broadening of our NCs, Raman spectroscopy was also used to characterize the CdS nanoparticles. All materials studied displayed two peaks centred *ca.* 300 cm^−1^ and 600 cm^−1^ (Fig. 2(c)). These are attributed to the two longitudinal optical (LO) modes of hexagonal CdS.^19,58^ Despite cubic CdS having a main LO peak centred *ca.* 350 cm^−1^, and the pXRD patterns suggesting the 3-FlAn-based systems were mainly cubic, this was not observed in any of our Raman spectra, indicating the likely presence of mixed phased synthesis in at least some of our proposed sphalerite systems.

Application of the Scherrer equation on the pXRD patterns found a range of sizes were present in the synthesised nanoscale CdS materials, with the TOPO only being the smallest (*ca.* 20 nm). Along the (002) reflection, the 5 : 1 TOPO : 3-FlBzAm was the largest (*ca.* 200 nm) and the 10 : 1 and 64 : 1 TOPO : 3-FlBzAm were calculated as *ca.* 80 nm. The 5 : 1 TOPO : 3-FlBzAm NCs along the (11̄0) and (11̄1) reflections were more comparable in size to the other two synthesised systems at *ca.* 80 nm, respectively, indicating rod shaped crystallites were synthesised (with an aspect ratio of *ca.* 2.5 : 1, *i.e.* 200 nm in the (002) direction and *ca.* 80 nm in the (11̄0) and (11̄1) directions). Overlapping peaks precluded this aspect analysis for the 10 : 1 and 64 : 1 TOPO : 3-FlBzAm systems. The predominantly sphalerite 5 : 1 and 1 : 1 TOPO : 3-FlAn NCs were calculated as *ca.* 35 nm and *ca.* 70 nm, respectively, slightly smaller than the wurtzite phased 3-FlBzAm NCs, which is consistent with previous reports indicating that larger nanoparticles preferentially form wurtzite over sphalerite.^28^

**Fig. 3:**
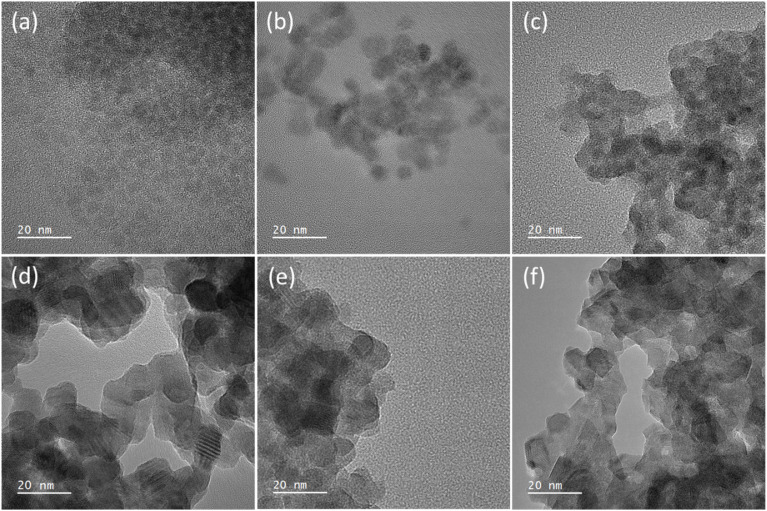
High resolution TEM images of the synthesised nanocrystals where (a) is TOPO only, (b) 5 : 1 and (c) 1 : 1 TOPO : 3-FlAn and (d) 5 : 1, (e) 10 : 1 and (f) 64 : 1 of TOPO : 3-FlBzAm, respectively. All scale bars are set to 20 nm. Ratio of TOPO : fluorinated amine are by mass, as in Table 1.

Transmission electron microscopy (TEM) was used to image the synthesised nanocrystals to further assess the particle size. Fig. 3 shows the images, which found that for (a–c) the TOPO only and the two 3-FlAn-based systems are discrete and roughly spherical particles were obtained of sub-10 nm in size. The 3-FlBzAm-based systems were found to be more complex, in these systems the NCs appeared to aggregate more strongly than in the 3-FlAn reactions. This is consistent with their predominant phases, as wurtzite CdS NCs have been shown to aggregate more strongly than sphalerite.^59^ This aggregation precluded any size analysis on the 10 : 1 and 64 : 1 TOPO : 3-FlBzAm systems but the 5 : 1 TOPO : 3-FlBzAm NCs were within the 50–100 nm range, consistent with the Scherrer analysis of the pXRD patterns. Aggregation could be expected due to the small size of the fluorinated aromatic amines, allowing the NCs to come into close contact; this can be a desired property as small, conductive surface-bound species are known to significantly facilitate electrocatalysis.^60,61^ CdS NCs synthesized in TOPO were characterized by scanning transmission electron microscopy (STEM). STEM bright-field (STEM-BF) micrographs taken from these CdS NCs (Fig. S1†) can be compared to the corresponding HR-TEM image (Fig. 3(a)). The STEM-BF image presents improved contrast compared to the HR-TEM and highlights the irregular shape of the NCs; elemental mapping was also carried out (Fig. S2†). STEM-EDS spectra of three different areas in the sample were analysed (Fig. S2†): CdS NCs (area 1), the supporting carbon film (area 2) and vacuum (area 3). Compared to the empty support carbon film area, the area containing CdS NCs presents stronger O (k_α_) signals. Additionally, phosphorus was detected near the CdS NCs and not on the supporting carbon films. These results all suggest the binding of TOPO to the NCs.

### Characterisation of CdS NCs with solid-state NMR

**Fig. 4:**
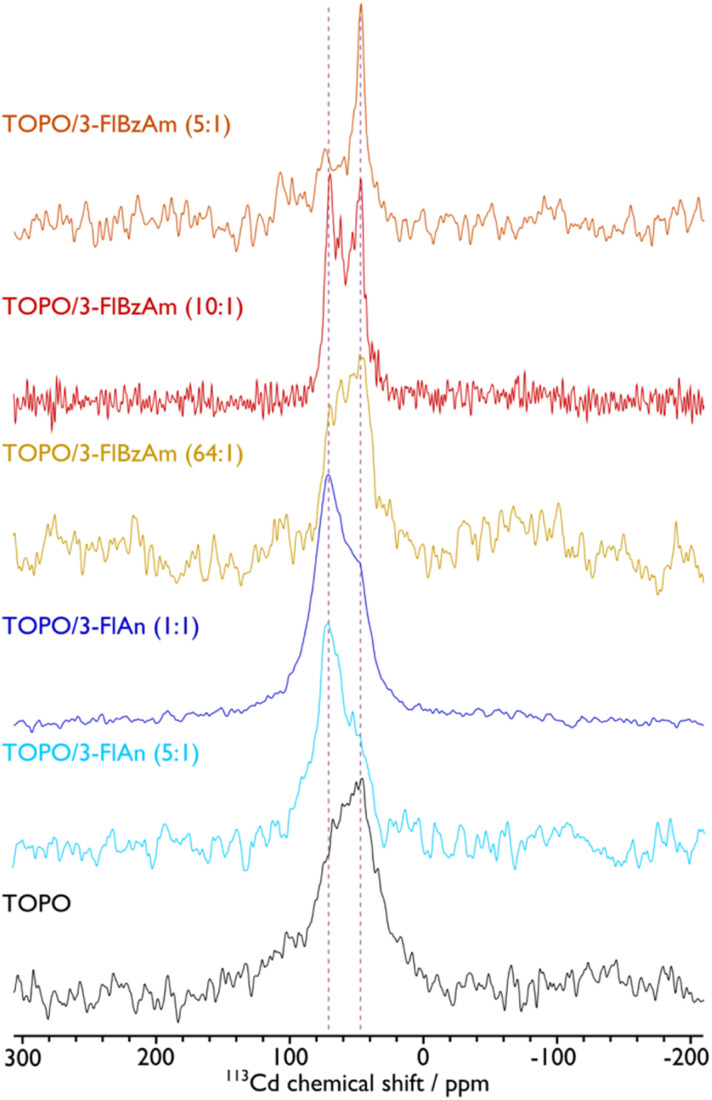
^113^Cd MAS NMR spectra of CdS NCs with ligands indicated. A MAS frequency of 8 kHz was used for TOPO, 10 kHz was used for TOPO/3-FlAn (1 : 1), and 12 kHz was used for TOPO/3-FlAn (5 : 1), TOPO/3-FlBzAm (64 : 1), TOPO/3-FlBzAm (10 : 1), and TOPO/3-FlBzAm (5 : 1) CdS NCs. Vertical dashed lines highlight peak positions for wurtzite (∼48 ppm) and sphalerite (∼71 ppm) CdS NCs. See the ESI† for corresponding spectral fittings. Ratio of TOPO : fluorinated amine are by mass, as in Table 1.

The presence of hexagonal CdS from Raman spectroscopy in all systems and cubic CdS from pXRD analysis for the FlAn-based CdS nanocrystals suggests the presence of both phases in the synthesised materials. To examine this further, solid-state NMR spectroscopy was used to assess the ^113^Cd environments within the synthesised nanocrystals. Fig. 4 shows the corresponding ^113^Cd magic angle spinning (MAS) NMR spectra for the CdS NCs studied here. The TOPO-only NCs, which have the smallest average particle size, exhibit a broad distribution of ^113^Cd chemical shifts ranging approximately from 0 to 120 ppm. This distribution cannot be fitted with a single Gaussian lineshape (see the ESI† for non-definitive deconvolutions, and Fig. S4† for TOPO CdS NCs). Therefore, this suggests that there are a variety of Cd environments, possibly from different CdS phases, and the shape of the spectrum suggests those that provide lower chemical shifts are preferred and in the majority. Different phases can be expected as a mixture,^37^ and also within individual particles due to stacking faults that arise at the synthetic stage.^44^ Although the ^113^Cd MAS NMR spectra of TOPO/3-FlAn CdS NCs are also broad and span a similar range, they differ from that of the TOPO-only NCs in that there is a clear majority of Cd environments that exhibit a higher chemical shift. The TOPO/3-FlBzAm CdS NCs also provide a distribution of ^113^Cd chemical shifts. However, as the proportion of 3-FlBzAm ligand increases, narrower ^113^Cd peaks begin to appear and there is an evident single dominating peak at *δ*{^113^Cd} = 48 ppm for the highest proportion (TOPO/3-FlBzAm (5 : 1); see also Fig. S10†). Combining with the pXRD analysis (*vide supra*), which showed that TOPO/3-FlBzAm CdS NCs were predominantly a hexagonal (wurtzite) phase and that TOPO/3-FlAn CdS NCs were a cubic (sphalerite) phase, lower ^113^Cd chemical shifts (*δ*{^113^Cd} < ∼60 ppm) can be tentatively assigned to wurtzite-CdS NCs and higher ^113^Cd chemical shifts (*δ*{^113^Cd} > ∼60 ppm) can be tentatively assigned to sphalerite-CdS NCs. This is consistent with complementary studies of CdSe NCs that have shown that core ^113^Cd chemical shifts are lower for wurtzite-CdSe NCs (*δ*{^113^Cd} = −80 ppm) than for sphalerite-CdSe NCs (*δ*{^113^Cd} = −66 ppm).^62^ The narrow ^113^Cd resonance observed for TOPO/3-FlBzAm (5 : 1) CdS NCs indicates a high-level of crystallinity and/or smaller distribution of particle sizes and is consistent with the pXRD analysis, which provided a dimension of ∼200 nm for the (002) reflection for these NCs whereas all others had all dimensions <100 nm. There are no spinning side bands visible in the ^113^Cd MAS NMR spectra. This indicates that the ^113^Cd environments have high tetrahedral symmetry and thus that the resonances arise from core (not surface) species.^63^ No other ^113^Cd resonances are observed for the CdS NCs prepared with fluorinated aromatic amines. However, a peak at *δ*{^113^Cd} = −650 ppm, which can be assigned to cadmium oxide impurities,^63^ can be seen for the TOPO-only NCs (see Fig. S4†). The presence of undecomposed precursor in the resulting powder was excluded, as the ^113^Cd NMR spectra of the precursor indicated a peak around *δ*{^113^Cd} = −447 ppm (see Fig. S3†). The breadth of the NMR peak at *δ*{^113^Cd} = −650 ppm and the lack of corresponding peaks in the pXRD pattern suggests that this impurity may be an amorphous CdO_*x*_ phase or CdO_*x*_ shell in the CdS NCs. This result correlates well with the STEM-EDS results, which suggest that the TOPO-only CdS NCs contain a substantial amount of oxygen (*vide supra*). Therefore, the fluorinated aromatic amines not only play a role in controlling the structure of the CdS NCs,^64^ but they also perform a stabilising role that prevents oxidation.

The ^113^Cd NMR data show that the inorganic component of the NCs is influenced by the ligands present during their synthesis. ^13^C NMR spectroscopy can be used to probe the organic, surface component. The ^13^C MAS NMR spectra, acquired using cross-polarization (CP) from ^1^H spins, show ^13^C resonances for both the TOPO and the fluorinated aromatic amine ligands in the respective samples (see Fig. S11†). The use of CP will favour ligands that are not highly dynamic and thus both sets of ligands are likely anchored to the NC surface. Interestingly, for the mixed-ligand NCs the ^13^C resonances from TOPO differ slightly to that from the TOPO-only CdS NCs. This indicates that the fluorinated aromatic amine ligands perturb the environment of the TOPO ligands. Moreover, whereas the ^13^C resonances from the TOPO and 3-FlBzAm ligands are relatively narrow, those from 3-FlAn ligands are broad. Thus, the 3-FlAn ligands are present in a wide range of geometries on the NC surfaces.

Since {^1^H–}^13^C CPMAS NMR spectra are not intrinsically quantitative and the experimental sensitivity is relatively poor, ^1^H MAS NMR spectra were also recorded so that the final ratio of TOPO to fluorinated aromatic amine could be determined. Moreover, the ^1^H MAS NMR spectra show that there is negligible residual (un-bound) ligand remaining as this would present discrete narrow peaks owing to greater ligand mobility.^65^ Even though the resolution of solid-state ^1^H MAS NMR under the employed experimental conditions is usually relatively poor, the spectra could be deconvoluted and integrated (see Fig. S12–S17 and Table S2†). The signal intensities of the aromatic protons from 3-FlAn and 3-BzAm were compared to those from the aliphatic protons from the TOPO ligand. This gave molar ratios of 1.6 : 1, 1 : 4.7, 1 : 2.7, 1 : 7, and 1 : 6.3 for TOPO : 3-FlAn (5 : 1), TOPO : 3-FlAn (1 : 1), TOPO : 3-FlBzAm (64 : 1), TOPO : 3-FlBzAm (10 : 1), and TOPO : 3-FlBzAm (5 : 1), respectively; the molar ratios added during the synthesis were 1.5 : 1, 1 : 3.5, 16 : 1, 3 : 1, and 1.5 : 1, respectively. Therefore, the resulting relative amount of 3-FlAn ligand is similar to that at the synthesis stage and the competition for surface adsorption is balanced between this ligand and TOPO. Contrastingly, the final amount of 3-FlBzAm on the CdS NCs vastly exceeds that of TOPO, highlighting that 3-FlBzAm has a much greater surface affinity. The final molar ratio seems to have plateaued at ∼1 : 6.5 TOPO : 3-FlBzAm. The greater surface affinity of the 3-FlBzAm ligand is likely to provide higher surface stability. It could be postulated that the methylene group of 3-FlBzAm provides extra structural flexibility, compared to 3-FlAn, allowing a more favourable surface arrangement of the ligands. This would be consistent with the ^13^C NMR data (*vide supra*), which shows that 3-FlBzAm is more ordered than 3-FlAn on the CdS NC surfaces.

**Fig. 5:**
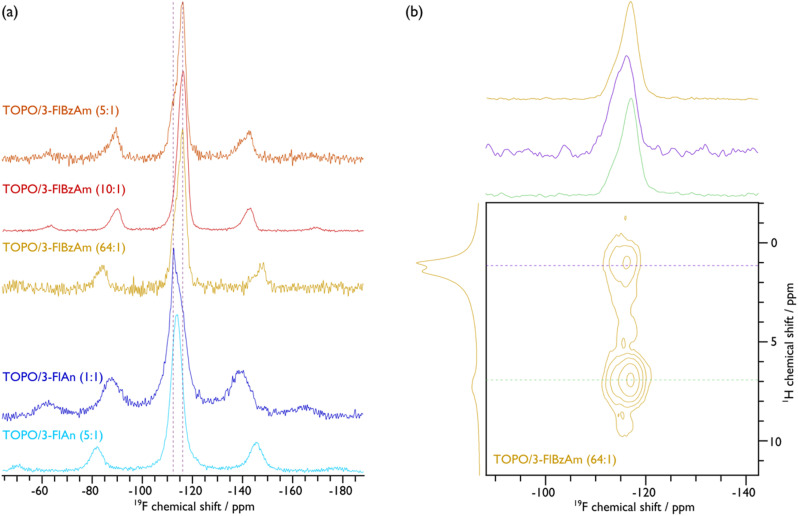
{^1^H–}^19^F CP (a) and ^1^H–^19^F 2D dipolar correlation (b) MAS NMR spectra of indicated CdS NCs. An MAS frequency of 10 kHz was used for TOPO/3-FlAn (1 : 1), TOPO/3-FlBzAm (10 : 1), and TOPO/3-FlBzAm (5 : 1), and 12 kHz was used for TOPO/3-FlAn (5 : 1) and TOPO/3-FlBzAm (64 : 1) CdS NCs. Cross-sectional slices at the positions indicated with the horizontal dashed coloured lines in the 2D spectrum are given above the 2D spectrum in (b) along with the corresponding {^1^H–}^19^F CP (top) and ^1^H (left) MAS NMR spectra. See the ESI† for corresponding spectral fittings.

To further investigate the ligand structure on the surface of the NCs, ^19^F MAS NMR spectroscopy was employed. The corresponding spectra, given in Fig. 5(a), display broad peaks with spinning sidebands for all samples, which provides further evidence that the fluorinated aromatic amines are surface-bound and are also not undergoing fast reorientations, as this would average the chemical shift anisotropy (see Fig. S15–S19 for fittings and Table S3† for the corresponding extracted parameters). That said, the TOPO/3-FlAn (1 : 1) CdS NCs also exhibit a narrow ^19^F resonance that does not have any measurable chemical shift anisotropy and can thus be attributed to 3-FlAn ligands that undergo fast isotropic motion, likely a result of free-ligand species that arise owing to their relatively high proportion in this sample. While there is only a single broad ^19^F resonance for the TOPO/3-FlAn CdS NCs, two overlapping broad resonances are observed for the TOPO/3-FlBzAm counterparts. Therefore, there are (at least) two environments for the 3-FlBzAm ligands on the CdS NCs. To gain further insight on these environments, a 2D ^1^H–^19^F dipolar correlation MAS NMR spectrum of TOPO/3-FlBzAm CdS NCs, shown in Fig. 5(b), was recorded. This experiment gives cross peaks (contours) between ^1^H and ^19^F nuclei that are in close spatial proximity. The large (^19^F, ^1^H; ppm) contour at (−116, 7) corresponds to a dipolar interaction between ^19^F and ^1^H from the 3-FlBzAm ligands themselves, as expected. Notably, there is also a substantial cross peak at (−115, 1) that highlights proximity between ^19^F from the 3-FlBzAm ligands and ^1^H from the TOPO ligands; a similar cross peak is also observed for TOPO/3-FlAn CdS NCs (see Fig. S23†). Accordingly, this infers that there is substantial ligand mixing on the surface of the NCs. This is in agreement with the ^13^C NMR data that shows that the TOPO resonances are perturbed with the addition of the amine-based ligands (*vide supra*). The centre of mass of the (−115, 1) cross peak is at higher (more positive) chemical shift than that of the (−116, 7) cross peak (see cross-sectional slices in Fig. 5(b)) and therefore the contribution at higher chemical shift will stem from 3-FlBzAm ligands that are closer to (more) TOPO ligands.

To determine the surface coverage of the fluorinated aromatic amine ligands, ^19^F–^19^F internuclear distances were probed using 1D double-quantum-filtered dipolar correlation MAS NMR spectra. The lattice constant for cubic (sphalerite) CdS is 5.8 Å and for hexagonal (wurtzite) CdS they are *a* = *b* = 4.2 and *c* = 6.8 Å. The Cd–S bond lengths are 2.6 Å in both phases. Plotting the relative intensity of double-quantum-filtered ^19^F signal as a function of dipolar mixing time and comparing to numerical simulations (given in Fig. S21†) shows that the ^19^F–^19^F internuclear distances can be <5 Å. There is likely a distribution of ^19^F–^19^F distances, but the initial slope of the plots supports the inference that the amine ligands can be 4.0–4.5 Å apart, which is consistent with the homoatomic distances (*e.g.* Cd–Cd or S–S; *cf.* 4.12 Å in sphalerite and 4.15 Å in wurtzite for both) and with recent investigations into the surface density of amine ligands on ZnS NCs.^66^ This means that the amine ligands can be bound at adjacent sites, providing good surface coverage. If multi-ligand interactions are accounted for (*i.e.*, three or more ^19^F homonuclear dipolar couplings, rather than spin pairs owing to the surface coverage) then the measured ^19^F–^19^F distance can be <4 Å. Hence, either the aromatic rings are rotated on the surface so that the Fs are pointing towards each other, the surface coverage is not monolayer (*i.e.* <100%), or the amines are bound at both Cd and S surface sites, if the terminating facet allows. The latter interpretation has been proposed based on ^15^N NMR and corresponding DFT calculations of amine ligands on CdSe NCs.^42^ As an inter-ligand distance of 4.2 Å is fully consistent with the interatomic spacings on both polar and non-polar CdS sphalerite and wurtzite facets, this regular spacing would also provide the opportunity for an ordered ligand arrangement, which is inferred from the ^13^C MAS NMR data of the majority wurtzite TOPO/3-FlBzAm CdS NCs (*vide supra*). However, for the majority sphalerite TOPO/3-FlAn CdS NCs that appear to have closer amine ligand packing (shorter ^19^F–^19^F distances (∼4.0 Å), see Fig. S24†) than with 3-FlBzAm, the greater extent of mixing with TOPO ligands leads to a disordered surface of 3-FlAn ligands, as inferred from the ^13^C MAS NMR data (*vide supra*). Nevertheless, the measured ^19^F–^19^F distances for both amine ligands demonstrates that a high surface coverage is achievable, which provides good surface passivation.

### Characterisation of CdS NC surfaces with X-ray photoelectron spectroscopy

With the characterisation of the CdS NCs indicating the presence of multiple phases and the solid-state NMR suggesting the presence of some amorphous CdO_*x*_ in the TOPO only sample, we undertook further surface characterisation using X-ray photoelectron spectroscopy (XPS). Survey spectra, Cd 3d, and S 2p are all shown in Fig. S22–S24.† From this analysis, a single chemical environment was found for all Cd 3d spectra and a single chemical environment for all S 2p spectra, both indicating a single CdS environment. Since it is challenging to distinguish between CdS and CdO with Cd 3d,^67^ O 1s was used to corroborate that the TOPO only NCs contain CdO_*x*_ as the associated relative intensity is much larger for these (see Fig. S25†). Moreover, quantitative analysis of Cd 3d and S 2p confirmed a 1 : 1 Cd : S ratio for all samples apart from the TOPO only system, which has a smaller amount of S (see Table S4†). The Cd MNN also confirms that no Cd^0^ is present.

Given the presence of fluorinated aromatic amine and TOPO capping agents, P 2p, N 1s, and F 1s were also interrogated. F 1s is possibly present is some of the samples, but not with sufficient concentration to be certain, N 1s could not be observed in any sample. P 2p was clearly observed in the survey spectra of the TOPO only and 5 : 1 TOPO : 3-FlAn synthesised systems (indicated in survey Fig. S22(a)†), but could not be found in other samples with any degree of certainty, this mirrors the quantitative trend observed in the ssNMR analysis where the 5 : 1 TOPO : 3-FlAn NCs was the only system that observed greater TOPO than fluorinated amine, all other samples had a higher degree of fluorinated amine than TOPO, which could explain why we struggled to observe further P in these samples.

### Absorbance and band gap analysis of CdS NCs

**Fig. 6:**
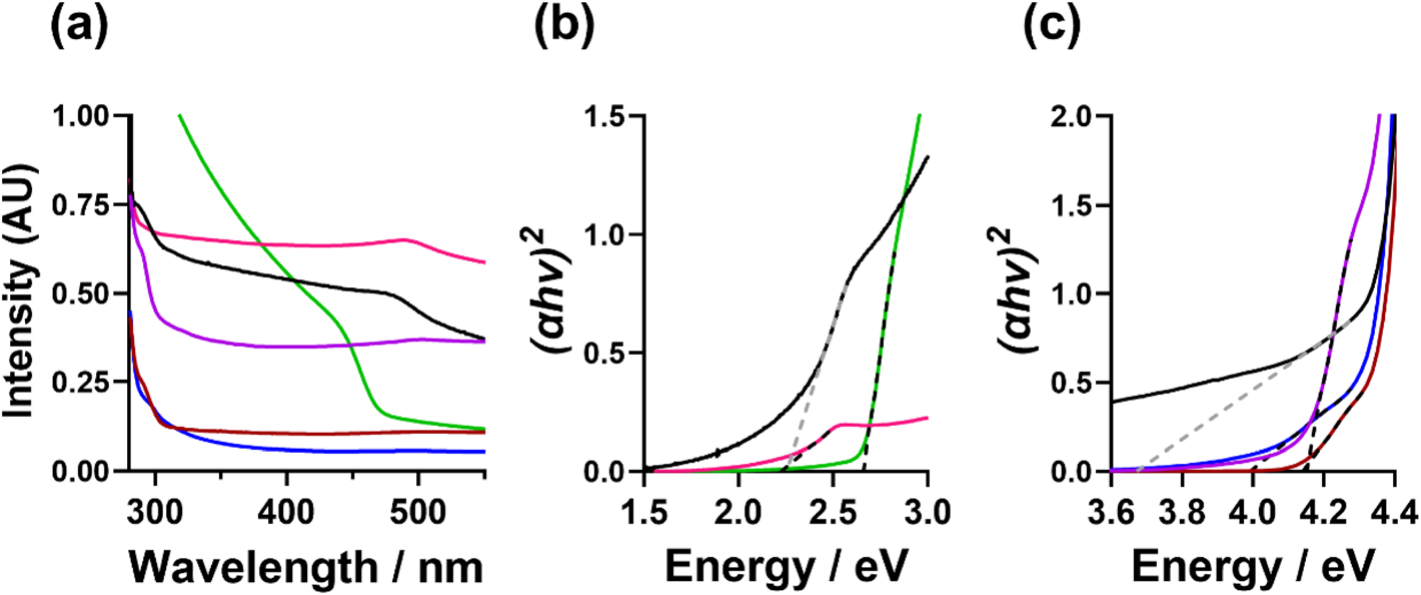
(a and b) UV-Vis absorption spectra and (c) calculated Tauc plots of the CdS nanocrystals suspended in toluene, where TOPO only (green), 5 : 1 TOPO : 3-FlAn (pink), 1 : 1 TOPO : 3-FlAn (black), 5 : 1 TOPO : 3-FlBzAm (blue), 10 : 1 TOPO : 3-FlBzAm (red) and 64 : 1 TOPO : 3-FlBzAm (purple) are all shown.

The optical absorption properties of the CdS NCs were measured by UV-Vis absorbance spectroscopy. Fig. 6(a) shows the UV-Vis absorption spectra and (Fig. c, d, and S25†) the resulting Tauc plots calculated to determine the band gap energies (*E*_g_). From this analysis, the TOPO-only, FlAn-based nanocrystals, and the 64 : 1 3-FlBzAm were found to have an absorption between 450 and 500 nm, with corresponding *E*_g_ of 2.7 eV (TOPO only), 2.2 eV (5 : 1 TOPO : FlAn) and 2.3 eV (1 : 1 TOPO : FlAn), and 2.2 (64 : 1 TOPO : 3-FlBzAm). The 1 : 1 3-FlAn, and 3-FlBzAm-based nanocrystals were found to have absorption at lower wavelengths (higher energies), between 280 and 310 nm, corresponding to *E*_g_ of 3.7 eV (1 : 1 TOPO : FlAn), 4.0 eV (3 : 2 TOPO : 3-FlBzAm), 4.1 eV (3 : 1 TOPO : 3-FlBzAm) and 4.2 eV (16 : 1 TOPO : 3-FlBzAm). Bulk, cubic CdS has a band gap energy *ca.* 2.4 eV^18,19^ and nanoparticulate hexagonal CdS has been reported with *E*_g_ in the range of 2.5–3.3 eV.^28,68^ A previous study of cubic and hexagonal CdS nanoparticles prepared through a solvothermal reaction of cadmium chloride and thiourea has found that hexagonal CdS nanoparticles have a *E*_g_ of *ca.* 2.5 eV, while cubic CdS nanoparticles were found to possess *E*_g_ of *ca.* 3.6 eV.^28^ In our nanocrystals, we observe the opposite trend where the cubic CdS nanocrystals possess lower band gap energies and hexagonal higher, with two of our systems having two band gap energies, which is likely linked to both phases being present.

The fact that our experimental findings differ from other reported CdS NCs indicates that the photon absorption properties of our samples are likely not related to the core CdS and could be related to the surface and the interaction with the surface ligands. The differing electronic effects of the amine group between the 3-FlBzAm and the 3-FlAn and the differing affinity of the different ligands could also influence the band structure of the surface CdS, along with possible defects, particle size, morphology, and strain within the lattice. This conclusion is supported by the ssNMR analysis indicating that FlBzAm has a higher affinity for CdS than FlAn, and the band gap energies of the TOPO only and TOPO/FlAn mixed systems possessing an *E*_g_ of *ca.* 2.5 eV and the TOPO/FlBzAm mixed systems possessing a *E*_g_ of *ca.* 4.0 eV. However, significant further work would be required in order to fully understand the electronic interaction between the surface ligands and CdS, and the localisation of photon absorption.

## Discussion

**Fig. 7:**
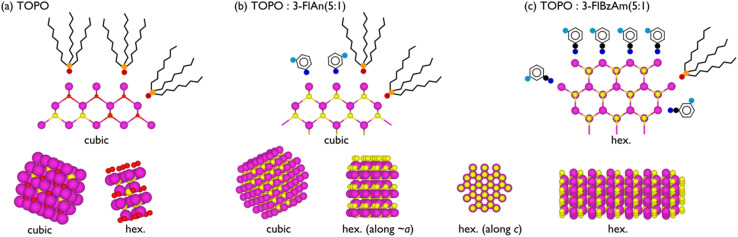
Schematic of (a) TOPO only, (b) 3-fluoroaniline : TOPO (1 : 5), and (c) 3-fluorobenzylamine : TOPO (1 : 5) CdS NCs. Purple, yellow, red, orange, blue, cyan, and black represent Cd, S, O, P, N, F, and C atoms, respectively. 2D slices illustrating surface ligand binding environments are given above 3D (not to scale) representations of the NC phases.

Here, solvent mixtures (aliphatic TOPO and mono-fluorinated aromatic amines) have been used to control the material properties of colloidal CdS nanocrystals. The TOPO-only CdS system was found to consist of small cubic and hexagonal CdS NCs (with the latter in the slight majority) that had a degree of amorphous CdO_*x*_. Using fluorinated amine : TOPO solvent mixtures prevented any oxidation, and the 3-fluorobenzylamine favoured the formation of (majority) hexagonal CdS NCs with a high surface binding affinity to CdS, whereas 3-fluoroaniline : TOPO mixtures were capable of stabilising the metastable cubic CdS polymorphs. Neither pXRD nor Raman spectroscopy were capable of clearly identifying that phase mixtures of CdS were present. ^113^Cd NMR was able to show that CdO_*x*_ was present in the TOPO only CdS NCs, and that both cubic and hexagonal CdS was present in all systems. ^13^C NMR spectra displayed broader resonances for 3-fluoroaniline species compared to the other two ligands, which indicates that they are disordered on the NC surface with negligible motion; the narrower lines observed for TOPO and 3-fluorobenzylamine, along with strong dipolar interactions, suggest that these ligands also display minimal motion but are ordered on the surface. The ^13^C NMR spectra, along with ^1^H–^19^F 2D dipolar correlation NMR spectra show that there is ligand mixing on the NC surfaces between TOPO and the mono-fluorinated aromatic amines. ^19^F–^19^F dipolar correlation NMR spectra indicate that this mixing is with a high surface coverage of ligands, likely with the amines binding to both Cd and S sites. The analysis herein is schematically represented in Fig. 7.

The stronger CdS binding affinity of 3-fluorobenzylamine compared to 3-fluoroaniline or TOPO could have possible implications on the optical absorption properties of the CdS NCs. The TOPO only and 3-fluoroaniline : TOPO mixtures all possessed a band gap energy *ca.* 2.5 eV, and the 3-fluorobenzylamine : TOPO CdS NCs displayed a higher band gap energy of *ca.* 4.0 eV. The ability to tune both phase and band gap of CdS has significant possible implications on photocatalysis using CdS NCs.

## Conclusions

Here, we have demonstrated the use of solid-state NMR spectroscopy to interrogate the role of fluorinated aromatic amine capping ligands in the formation of NPs using CdS as a model. The mono-fluorinated ligands combined with ^19^F ssNMR spectroscopy enabled the high surface coverage of ligands to be determined through ^19^F–^19^F internuclear distance measurements. A stronger binding affinity of 3-FlBzAm than 3-FlAn or TOPO to the CdS surface was found. This study also found that phase control of colloidal CdS nanocrystals using a mixed solvent system of TOPO : aromatic amine was possible. CdS nanocrystals synthesised from the TOPO : 3-FlAn regimes were predominantly cubic and the TOPO : 3-FlBzAm synthesised nanocrystals were predominantly hexagonal, although there is a mixture of phases present for all samples as determined from pXRD, Raman, and ssNMR analysis. It was also shown through ssNMR that the addition of aromatic amine was found to avoid CdO_*x*_ formation, which was observed for the TOPO-only regime. This work shows that combined aliphatic and aromatic solvent regimes can control the synthesised phase and band gap energy of CdS NCs, and that ssNMR is a powerful tool to interrogate both the nanoparticles and capping ligands of colloidal CdS NCs. The *E*_g_ determined through UV-Vis analysis shows that these NCs are good candidates as solar absorbers for either photovoltaic or photocatalytic applications.

## Data availability

Data for this article, including ssNMR data and TEM images, are available at Figshare at https://doi.org/10.48420/26098096.

## Conflicts of interest

There are no conflicts of interest to declare.

## Supplementary Material

NA-OLF-D4NA00564C-s001

## References

[cit1] Lin H., Chen Y., Shi J. (2018). Chem. Soc. Rev..

[cit2] Shin T.-H., Choi Y., Kim S., Cheon J. (2015). Chem. Soc. Rev..

[cit3] Saha K., Agasti S. S., Kim C., Li X., Rotello V. M. (2012). Chem. Rev..

[cit4] Gao C., Lyu F., Yin Y. (2021). Chem. Rev..

[cit5] Gawande M. B., Goswami A., Felpin F.-X., Asefa T., Huang X., Silva R., Zou X., Zboril R., Varma R. S. (2016). Chem. Rev..

[cit6] Alzahrani H. A. H., Buckingham M. A., Wardley W. P., Tilley R. D., Ariotti N., Aldous L. (2020). Chem. Commun..

[cit7] Alzahrani H. A. H., Buckingham M. A., Marken F., Aldous L. (2019). Electrochem. Commun..

[cit8] Stark W. J., Stoessel P. R., Wohlleben W., Hafner A. (2015). Chem. Soc. Rev..

[cit9] Kumar S. G., Rao K. S. R. K. (2014). Energy Environ. Sci..

[cit10] Gutierrez Z. B. K., Zayas-Bazán P. G., de Melo O., de Moure-Flores F., Andraca-Adame J. A., Moreno-Ruiz L. A., Martínez-Gutiérrez H., Gallardo S., Sastré-Hernández J., Contreras-Puente G. (2018). Materials.

[cit11] Chang C. M., Orchard K. L., Martindale B. C. M., Reisner E. (2016). J. Mater. Chem. A.

[cit12] Kuehnel M. F., Orchard K. L., Dalle K. E., Reisner E. (2017). J. Am. Chem. Soc..

[cit13] Kuehnel M. F., Wakerley D. W., Orchard K. L., Reisner E. (2015). Angew. Chem., Int. Ed..

[cit14] Wakerley D. W., Kuehnel M. F., Orchard K. L., Ly K. H., Rosser T. E., Reisner E. (2017). Nat. Energy.

[cit15] Wang Q., Warnan J., Rodríguez-Jiménez S., Leung J. J., Kalathil S., Andrei V., Domen K., Reisner E. (2020). Nat. Energy.

[cit16] Cheng L., Xiang Q., Liao Y., Zhang H. (2018). Energy Environ. Sci..

[cit17] Sun Q., Wang N., Yu J., Yu J. C. (2018). Adv. Mater..

[cit18] Buckingham M. A., Catherall A. L., Hill M. S., Johnson A. L., Parish J. D. (2017). Cryst. Growth Des..

[cit19] Buckingham M. A., Norton K., McNaughter P. D., Whitehead G., Vitorica-Yrezabal I., Alam F., Laws K., Lewis D. J. (2022). Inorg. Chem..

[cit20] Dabhane H., Ghotekar S., Tambade P., Pansambal S., Murthy H. C. A., Oza R., Medhane V. (2021). Environ. Chem. Ecotoxicol..

[cit21] Alderhami S. A., Ahumada-Lazo R., Buckingham M. A., Binks D. J., O'Brien P., Collison D., Lewis D. J. (2023). Dalton Trans..

[cit22] Nair P. S., Radhakrishnan T., Revaprasadu N., Kolawole G., O'Brien P. (2002). J. Mater. Chem..

[cit23] Sarker J. C., Hogarth G. (2020). Chem. Rev..

[cit24] Vogel W., Urban J., Kundu M., Kulkarni S. K. (1997). Langmuir.

[cit25] Zelaya-Angel O., Lozada-Morales R. (2000). Phys. Rev. B.

[cit26] Datta S., Saha-Dasgupta T., Sarma D. D. (2008). J. Phys.:Condens. Matter.

[cit27] Zelaya-angel O., Yee-madeira H., Lozada-morales R. (1999). Phase Transitions.

[cit28] Banerjee R., Jayakrishnan R., Ayyub P. (2000). J. Phys.:Condens. Matter.

[cit29] Mensah M. B., Awudza J. A. M., Revaprasadu N., O'Brien P. (2021). Mater. Sci. Semicond. Process..

[cit30] Sheng C. K., Alrababah Y. M. (2023). Heliyon.

[cit31] Fenton J. L., Steimle B. C., Schaak R. E. (2019). Inorg. Chem..

[cit32] Abdelhady A. L., Malik M. A., O'Brien P. (2014). J. Inorg. Organomet. Polym. Mater..

[cit33] Moloto M. J., Revaprasadu N., O'Brien P., Malik M. A. (2004). J. Mater. Sci.:Mater. Electron..

[cit34] Kandasamy K., Singh H. B., Kulshreshtha S. K. (2009). J. Chem. Sci..

[cit35] Azad Malik M., O'Brien P., Revaprasadu N. (2001). J. Mater. Chem..

[cit36] Srinivasan N., Thirumaran S. (2012). Superlattices Microstruct..

[cit37] Shen Q., Xue J., Mi A., Jia H., Liu X., Xu B. (2013). RSC Adv..

[cit38] Azimi H., Kuhri S., Osvet A., Matt G., Khanzada L. S., Lemmer M., Luechinger N. A., Larsson M. I., Zeira E., Guldi D. M., Brabec C. J. (2014). J. Am. Chem. Soc..

[cit39] Smith J. G., Jain P. K. (2016). J. Am. Chem. Soc..

[cit40] Heuer-Jungemann A., Feliu N., Bakaimi I., Hamaly M., Alkilany A., Chakraborty I., Masood A., Casula M. F., Kostopoulou A., Oh E., Susumu K., Stewart M. H., Medintz I. L., Stratakis E., Parak W. J., Kanaras A. G. (2019). Chem. Rev..

[cit41] Balitskii O. A., Sytnyk M., Stangl J., Primetzhofer D., Groiss H., Heiss W. (2014). ACS Appl. Mater. Interfaces.

[cit42] Cao W., Yakimov A., Qian X., Li J., Peng X., Kong X., Coperet C. (2023). Angew. Chem., Int. Ed..

[cit43] Chen Y., Dorn R. W., Hanrahan M. P., Wei L., Blome-Fernández R., Medina-Gonzalez A. M., Adamson M. A. S., Flintgruber A. H., Vela J., Rossini A. J. (2021). J. Am. Chem. Soc..

[cit44] Gao Y., Peng X. (2014). J. Am. Chem. Soc..

[cit45] Khan M. D., Opallo M., Revaprasadu N. (2021). Dalton Trans..

[cit46] Pradhan N., Reifsnyder D., Xie R., Aldana J., Peng X. (2007). J. Am. Chem. Soc..

[cit47] Lee D., Wolska-Pietkiewicz M., Badoni S., Grala A., Lewiński J., De Paëpe G. (2019). Angew. Chem..

[cit48] Badoni S., Terlecki M., Carret S., Poisson J.-F., Charpentier T., Okuno H., Wolska-Pietkiewicz M., Lee D., Lewiński J., De Paëpe G. (2024). J. Am. Chem. Soc..

[cit49] Dunlap-Shohl W. A., Tabassum N., Zhang P., Shiby E., Beratan D. N., Waldeck D. H. (2024). Sci. Rep..

[cit50] Zhou S., Gallant B. M., Zhang J., Shi Y., Smith J., Drysdale J. N., Therdkatanyuphong P., Taddei M., McCarthy D. P., Barlow S., Kilbride R. C., Dasgupta A., Marshall A. R., Wang J., Kubicki D. J., Ginger D. S., Marder S. R., Snaith H. J. (2024). J. Am. Chem. Soc..

[cit51] Vickers E. T., Graham T. A., Chowdhury A. H., Bahrami B., Dreskin B. W., Lindley S., Naghadeh S. B., Qiao Q., Zhang J. Z. (2018). ACS Energy Lett..

[cit52] Zhou D., Lin M., Chen Z., Sun H., Zhang H., Sun H., Yang B. (2011). Chem. Mater..

[cit53] Fung B. M., Khitrin A. K., Ermolaev K. (2000). J. Magn. Reson..

[cit54] Hohwy M., Rienstra C. M., Jaroniec C. P., Griffin R. G. (1999). J. Chem. Phys..

[cit55] Sowa H. (2005). Solid State Sci..

[cit56] Rodic D., Spasojevic V., Bajorek A., Omnerud P. (1996). J. Magn. Magn. Mater..

[cit57] Muniz F. T. L., Miranda M. A. R., Morilla Dos Santos C., Sasaki J. M. (2016). Acta Crystallogr., Sect. A:Found. Adv..

[cit58] Singh V., Sharma P. K., Chauhan P. (2010). Mater. Chem. Phys..

[cit59] Alevato V., Streater D., Huang J., Brock S. (2024). J. Phys. Chem. C.

[cit60] Kaminsky C. J., Weng S., Wright J., Surendranath Y. (2022). Nat. Catal..

[cit61] Ahn S., Klyukin K., Wakeham R. J., Rudd J. A., Lewis A. R., Alexander S., Carla F., Alexandrov V., Andreoli E. (2018). ACS Catal..

[cit62] Piveteau L., Ong T.-C., Walder B. J., Dirin D. N., Moscheni D., Schneider B., Bär J., Protesescu L., Masciocchi N., Guagliardi A., Emsley L., Copéret C., Kovalenko M. V. (2018). ACS Cent. Sci..

[cit63] Hanrahan M. P., Chen Y., Blome-Fernández R., Stein J. L., Pach G. F., Adamson M. A. S., Neale N. R., Cossairt B. M., Vela J., Rossini A. J. (2019). J. Am. Chem. Soc..

[cit64] Endres E. J., Bairan Espano J. R., Koziel A., Peng A. R., Shults A. A., Macdonald J. E. (2024). ACS Nanosci. Au.

[cit65] Hens Z., Martins J. C. (2013). Chem. Mater..

[cit66] Chen W., Xiao H., Zhang M., Wang C., Chen J., Mao R., Jiang L., Hsu H.-Y., Buntine M. A., Shao Z., Yang X., Li C., Rogach A. L., Jia G. (2024). J. Am. Chem. Soc..

[cit67] Li W., Li M., Xie S., Zhai T., Yu M., Liang C., Ouyang X., Lu X., Li H., Tong Y. (2013). CrystEngComm.

[cit68] Marandi M., Taghavinia N., Zad A. I., Mahdavi S. M. (2005). Nanotechnology.

