# Supplementary material for: Passivation, phase, and morphology control of CdS nanocrystals probed using fluorinated aromatic amines and solid-state NMR spectroscopy
Source: Nanoscale Adv. 2024 Dec 18;7(3):850–61. doi: 10.1039/d4na00564c (PMC11658078; doi:10.1039/d4na00564c)
Supplement: NA-007-D4NA00564C-s001 [file NA-007-D4NA00564C-s001.pdf]

Supplementary Information (SI) for Nanoscale Advances.
This journal is © The Royal Society of Chemistry 2024

Supporting information for:

Passivation, phase, and morphology control of CdS nanocrystals probed using fluorinated aromatic amines and solid-state NMR spectroscopy

Mark A. Buckingham,[a,§] Robert Crawford,[b,§] Yi Li,[a,§] Ran Eitan Abutbul,[b] Bing Han,[a] Kerry Hazledine,[c] Sarah Cartmell,[a] Alex Walton,[c] Alex S. Eggeman,[a] David J. Lewis*[a] and Daniel Lee*[b]

[a] Department of Materials, The University of Manchester, Manchester M13 9PL, U.K.

[b] Department of Chemical Engineering, University of Manchester, Manchester M13 9PL, UK.

[c] Department of Chemistry and the Photon Science Institute, The University of Manchester, Oxford Road, Manchester, M13 9PL, UK.

[§] These authors contributed equally

* Corresponding authors: David.lewis-4@manchester.ac.uk, Daniel.lee@manchester.ac.uk

Transmission electron microscopy

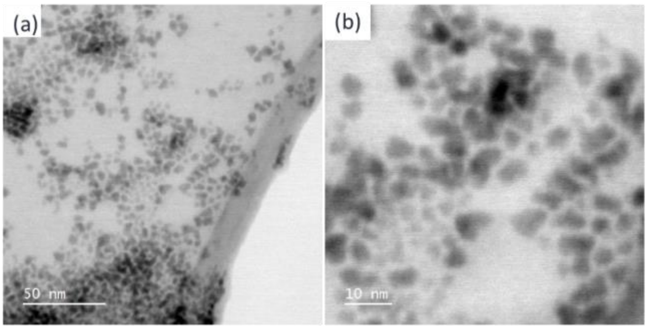

Figure S1. Scanning transmission electron microscope (STEM) bright field (BF) micrograph of CdS NCs synthesized in TOPO.

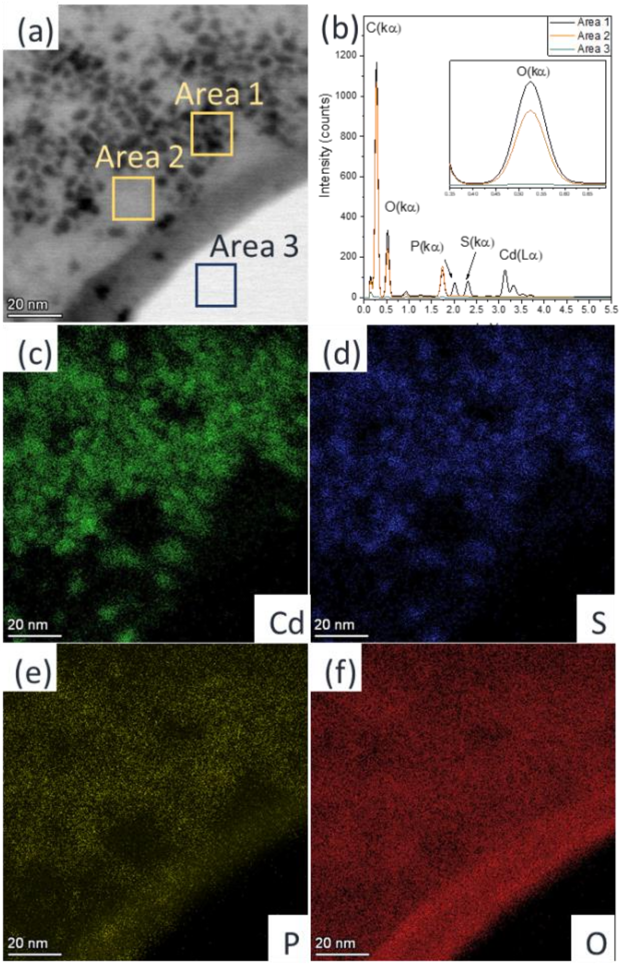

Figure S2. (a) Scanning transmission electron microscope (STEM) bright field (BF) micrograph of CdS NCs synthesized in TOPO (b) STEM-EDS spectra taken from area highlighted in a. Inset shows enlarged area of the O kα lines. The difference in the EDS spectra between area 1 and area 2 suggest higher content of O in the vicinity of the CdS NC. Elemental mapping of the image shown in a. (c) Cadmium (d) Sulphur (e) Phosphorous (f) Oxygen

Solid state NMR

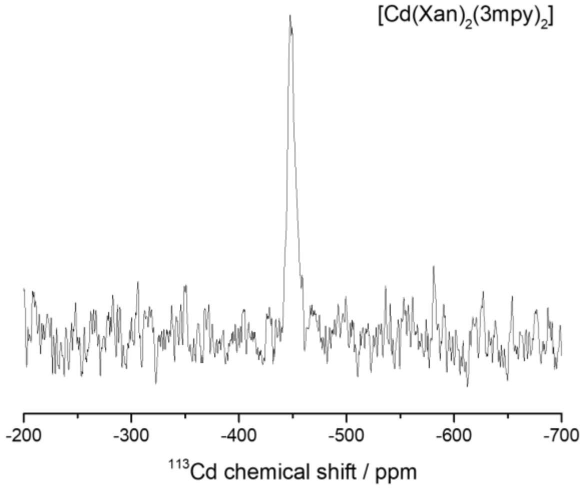

Figure S3. ^{113}Cd MAS NMR spectrum of the molecular precursor [Cd(Xan)$_2$(3-mpy)$_2$].

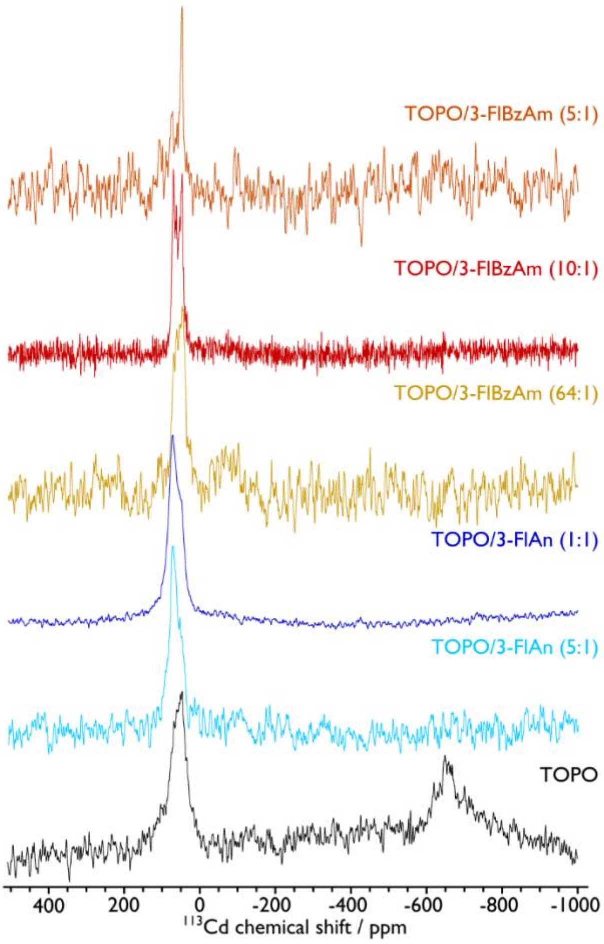

Figure S4.[113]Cd MAS NMR spectra of CdS NCs with indicated ligands. An MAS frequency of 8 kHz was used for TOPO, 10 kHz was used for TOPO/3-FlAn (1:1), and 12 kHz was used for TOPO/3-FlAn (5:1), TOPO/3-FlBzAm (64:1), TOPO/3-FlBzAm (10:1), and TOPO/3-FlBzAm (5:1) CdS NCs.

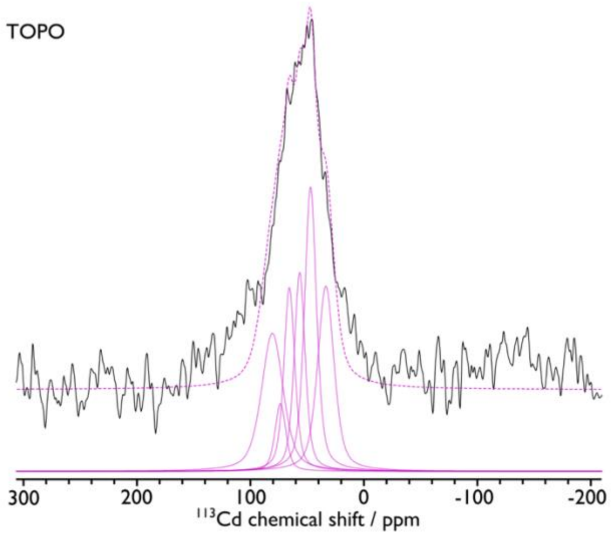

Figure S5. ^{113}Cd MAS NMR spectrum of TOPO CdS NCs and associated fitting with deconvolution (pink) and summation (dashed pink).

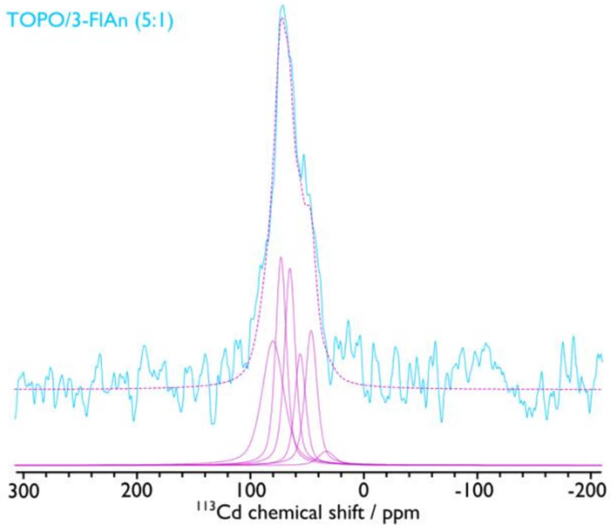

Figure S6. ^{113}Cd MAS NMR spectrum of TOPO/3-FlAn (5:1) CdS NCs and associated fitting with deconvolution (pink) and summation (dashed pink).

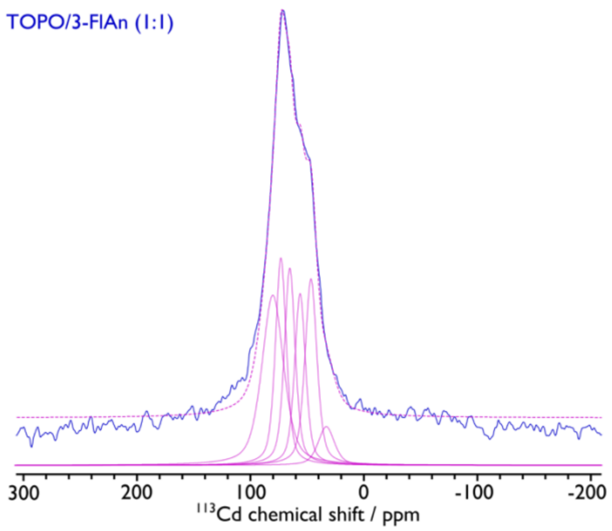

Figure S7. ^{113}Cd MAS NMR spectrum of TOPO/3-FlAn (1:1) CdS NCs and associated fitting with deconvolution (pink) and summation (dashed pink).

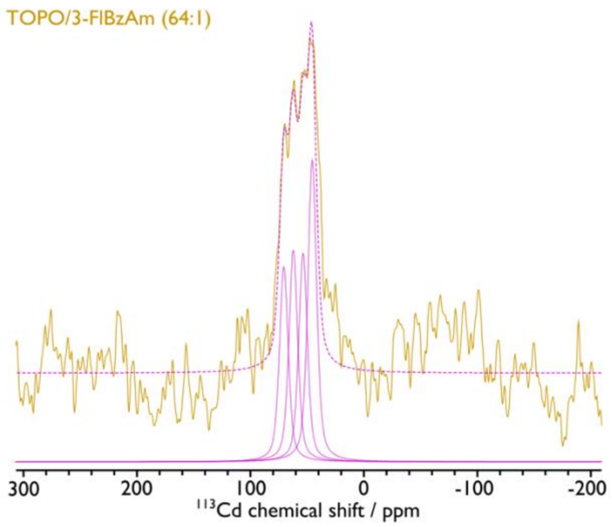

Figure S8. ^{113}Cd MAS NMR spectrum of TOPO/3-FlBzAm (64:1) CdS NCs and associated fitting with deconvolution (pink) and summation (dashed pink).

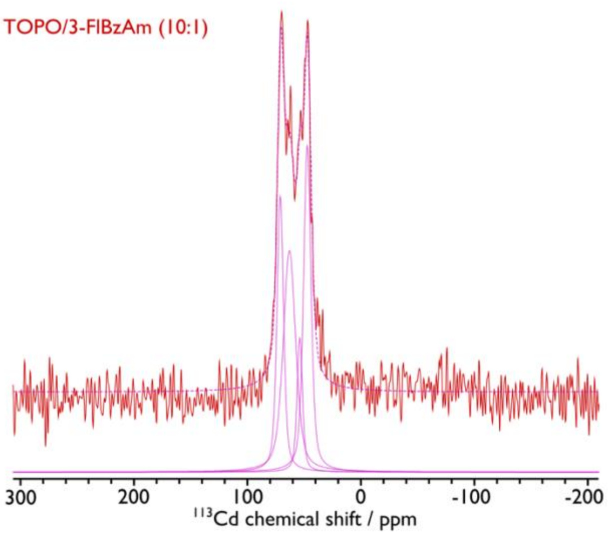

Figure S9. ^{113}Cd MAS NMR spectrum of TOPO/3-FlBzAm (10:1) CdS NCs and associated fitting with deconvolution (pink) and summation (dashed pink).

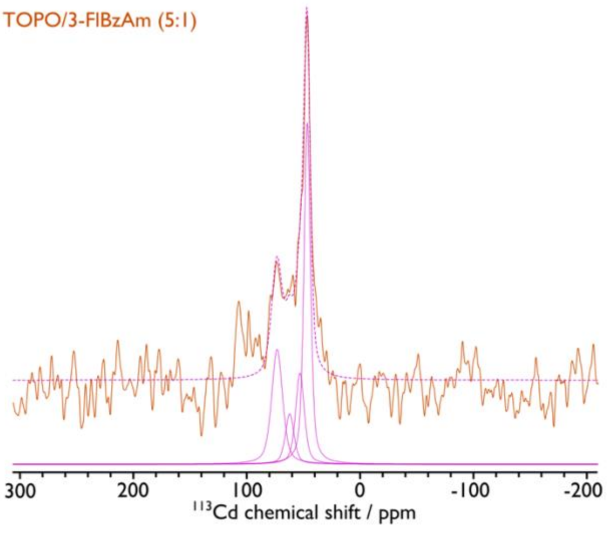

Figure S10. ^{113}Cd MAS NMR spectrum of TOPO/3-FlBzAm (5:1) CdS NCs and associated fitting with deconvolution (pink) and summation (dashed pink).

Table S1. ^{113}Cd NMR parameters extracted from data fitting (see Figs. S2-S7). The fits are not definitive owing to the large associated errors and ambiguity.

CdS nanocrystal system	^{19}F chemical shift (δ_{iso}) / ppm	^{19}F linewidth (FWHM) / Hz	Relative integral / %
TOPO only	34<br>48<br>57<br>66<br>74<br>81	1500<br>1100<br>1000<br>1000<br>1000<br>2000	21<br>24<br>15<br>14<br>5<br>21
TOPO:3-FlAn (5:1)	34<br>48<br>57<br>66<br>74<br>81	1500<br>1100<br>1000<br>1000<br>1000<br>2000	2<br>16<br>12<br>21<br>22<br>27
TOPO:3-FlAn (1:1)	34<br>48<br>57<br>66<br>74<br>81	1500<br>1100<br>1000<br>1000<br>1000<br>2000	5<br>17<br>15<br>17<br>17<br>29
TOPO:3-FlBzAm (64:1)	46<br>54<br>63<br>71	800<br>800<br>800<br>800	33<br>23<br>23<br>21
TOPO:3-FlBzAm (10:1)	48<br>54<br>63<br>71	680<br>505<br>1150<br>605	31<br>9<br>36<br>24
TOPO:3-FlBzAm (5:1)	48<br>54<br>63<br>74	600<br>800<br>800<br>1000	47<br>26<br>17<br>10

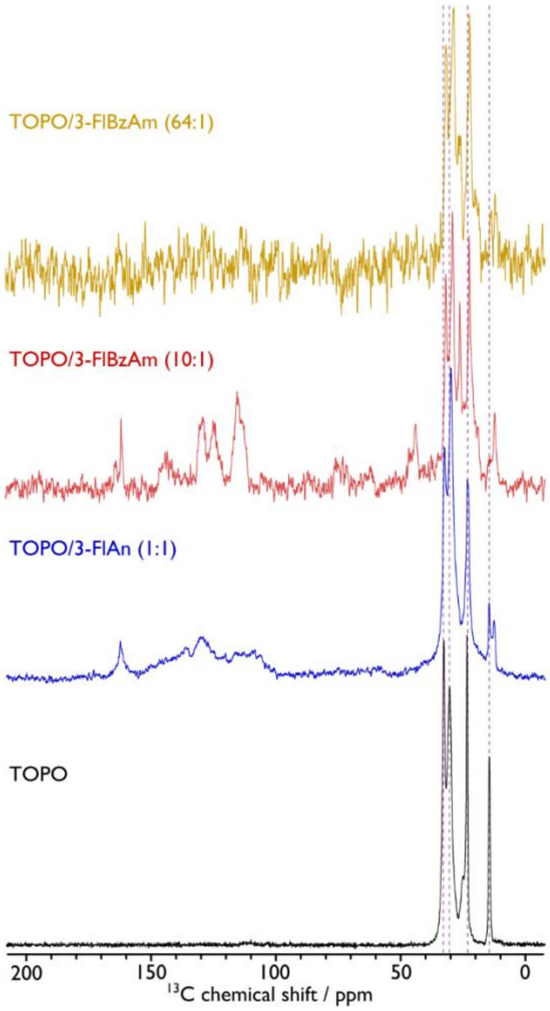

Figure S11. {1H-}^{13}C CPMAS NMR spectra. An MAS frequency of 8 kHz was used for TOPO, 10 kHz was used for TOPO/3-FlAn (1:1) and TOPO/3-FlBzAm (10:1), and 12 kHz was used for TOPO/3-FlBzAm (64:1) CdS NCs. The dashed vertical lines are a guide for the eye.

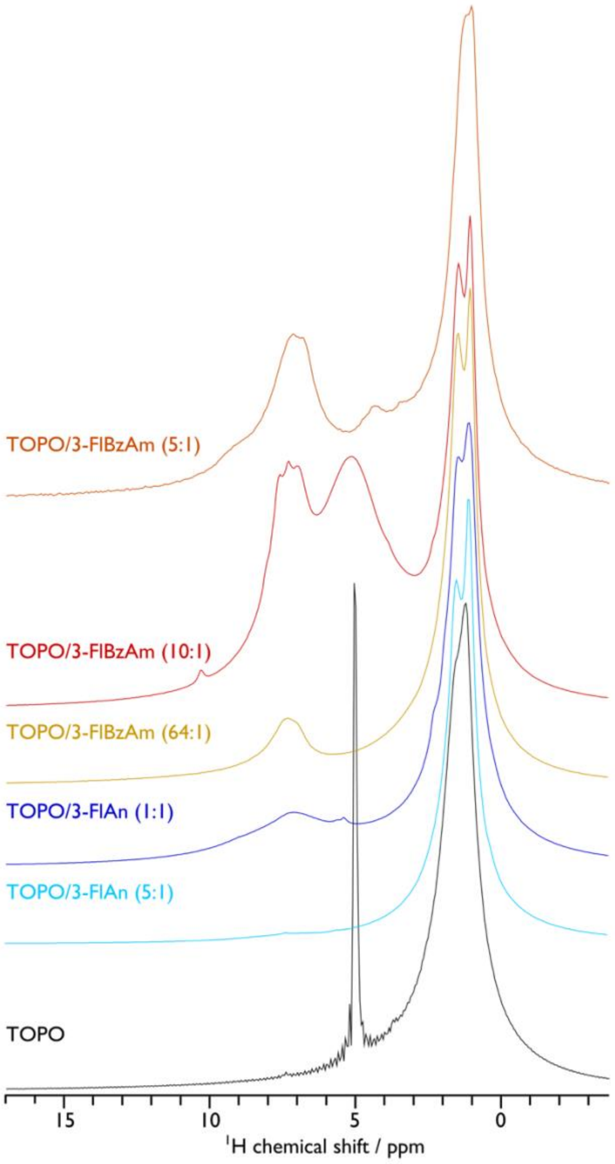

Figure S12. ^{1}H MAS NMR spectra. An MAS frequency of 10 kHz was used for TOPO/3-FlAn (1:1), TOPO/3-FlBzAm (10:1), and TOPO/3-FlBzAm (5:1), and 12 kHz was used for TOPO, TOPO/3-FlAn (5:1), and TOPO/3-FlBzAm (64:1) CdS NCs.

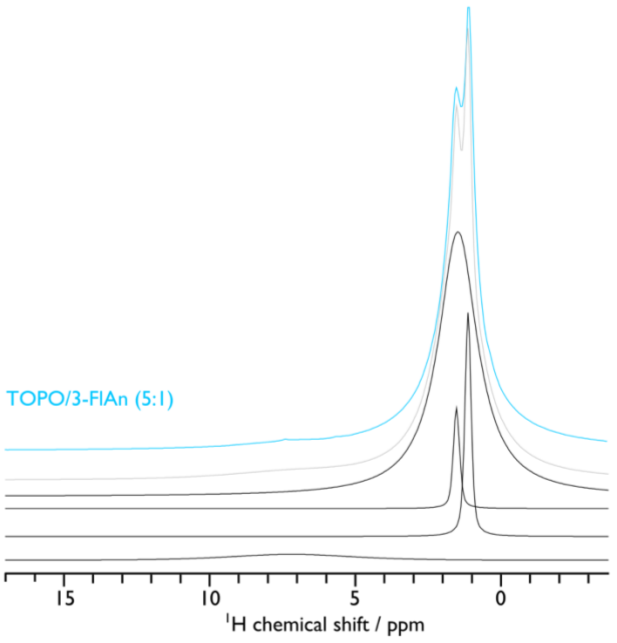

Figure S13. ¹H MAS NMR spectrum of TOPO/3-FlAn (5:1) CdS NCs and associated fitting with deconvolution (black) and summation (grey).

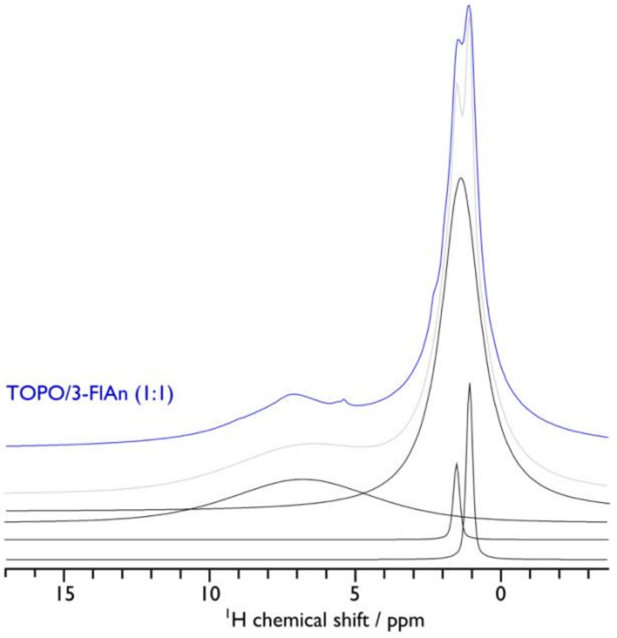

Figure S14. ¹H MAS NMR spectrum of TOPO/3-FlAn (1:1) CdS NCs and associated fitting with deconvolution (black) and summation (grey).

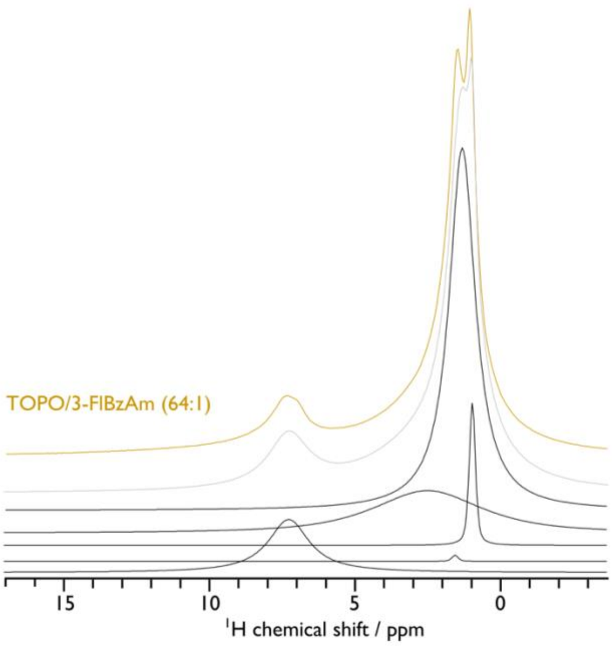

Figure S15. ¹H MAS NMR spectrum of TOPO/3-FlBzAm (64:1) CdS NCs and associated fitting with deconvolution (black) and summation (grey).

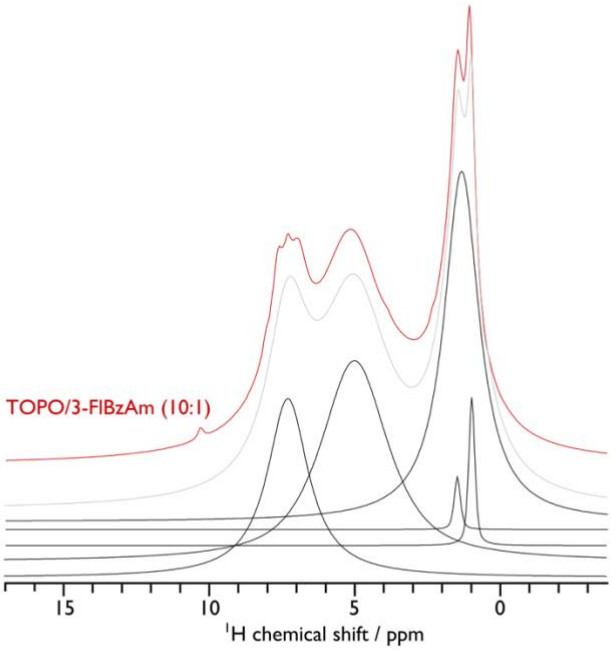

Figure S16. ¹H MAS NMR spectrum of TOPO/3-FlBzAm (10:1) CdS NCs and associated fitting with deconvolution (black) and summation (grey).

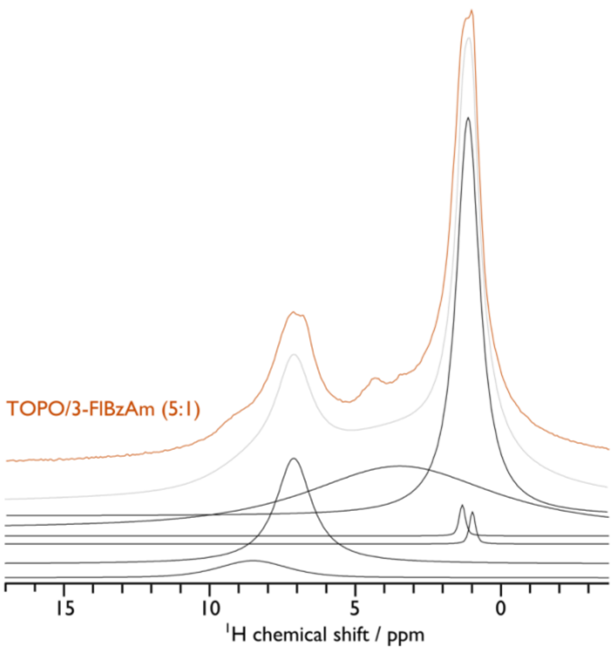

Figure S17. ¹H MAS NMR spectrum of TOPO/3-FlBzAm (5:1) CdS NCs and associated fitting with deconvolution (black) and summation (grey).

Table S2. ^{1}H NMR parameters extracted from data fitting (see Figs. S10-S14). The fits are not definitive owing to the large associated errors and ambiguity.

**CdS nanocrystal system**	**^{1}H chemical shift (δ_{iso}) / ppm**	**^{1}H linewidth (FWHM) / Hz**	**Relative integral / %**	**TOPO:ligand molar ratio (from ^{1}H integral)**	**Expected TOPO:ligand molar ratio**
TOPO only	N/A	N/A	N/A	N/A	N/A
TOPO:3-FlAn (5:1)	1<br>1.4<br>1.5<br>7.4	100<br>650<br>100<br>2000	9<br>82<br>4<br>5	1.6:1	1.5:1
TOPO:3-FlAn (1:1)	1<br>1.4<br>1.5<br>7.4	100<br>650<br>100<br>2300	4<br>67<br>2<br>27	1:4.7	1:3.5
TOPO:3-FlBzAm (64:1)	1<br>1.4<br>1.5<br>2.6<br>7.4	100<br>440<br>100<br>1800<br>680	4<br>57<br>0<br>26<br>13	1:2.7	16:1
TOPO:3-FlBzAm (10:1)	1<br>1.4<br>1.5<br>5.1<br>7.4	100<br>580<br>100<br>1100<br>680	2<br>37<br>1<br>38<br>22	1:7.0	3:1
TOPO:3-FlBzAm (5:1)	1<br>1.4<br>1.5<br>3.2<br>7.4<br>8.6	100<br>390<br>100<br>3000<br>600<br>1120	1<br>37<br>1<br>42<br>15<br>4	1:6.3	1.5:1

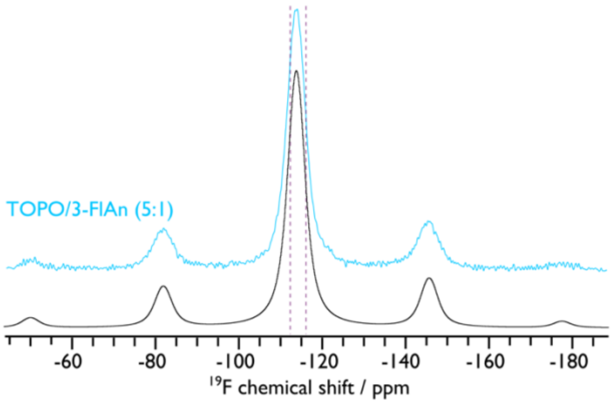

Figure S18. {^{1}H-}^{19}F CP MAS NMR spectrum of TOPO/3-FlAn (5:1) CdS NCs and associated fit (black). The positions indicated with the vertical dashed lines are the same as those given in Figure 5 of the main text.

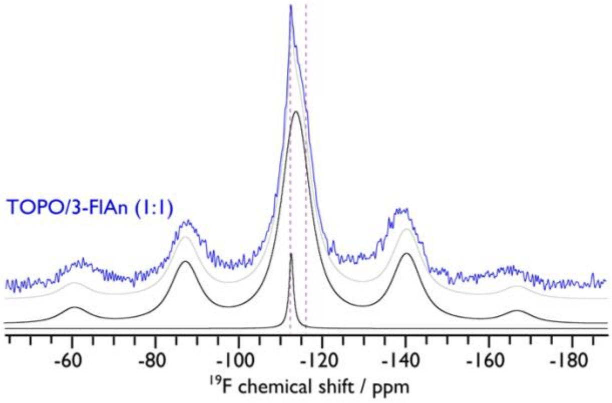

Figure S19. {^{1}H-}^{19}F CP MAS NMR spectrum of TOPO/3-FlAn (1:1) CdS NCs and associated fitting with deconvolution (black) and summation (grey). The positions indicated with the vertical dashed lines are the same as those given in Figure 5 of the main text.

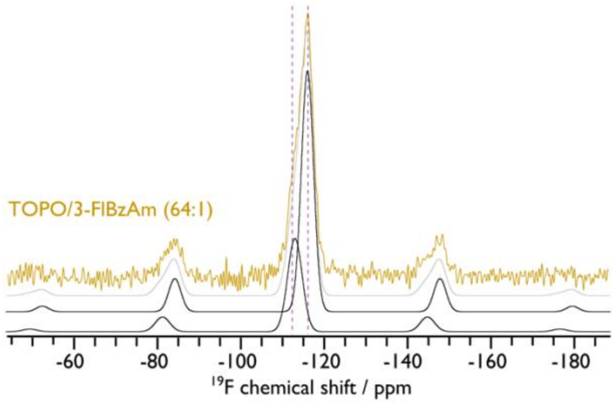

Figure S20. {^{1}H-}^{19}F CP MAS NMR spectrum of TOPO/3-FlBzAm (64:1) CdS NCs and associated fitting with deconvolution (black) and summation (grey). The positions indicated with the vertical dashed lines are the same as those given in Figure 5 of the main text.

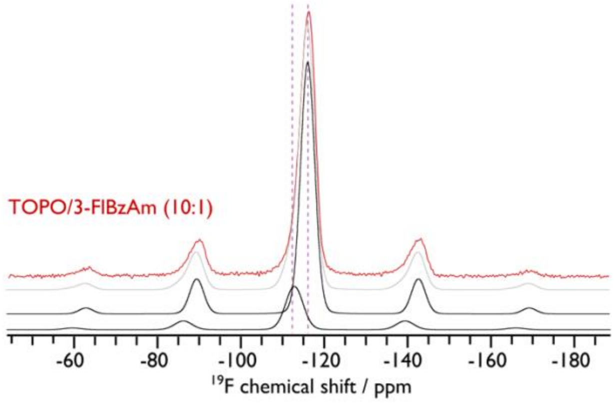

Figure S21. {^{1}H-}^{19}F CP MAS NMR spectrum of TOPO/3-FlBzAm (10:1) CdS NCs and associated fitting with deconvolution (black) and summation (grey). The positions indicated with the vertical dashed lines are the same as those given in Figure 5 of the main text.

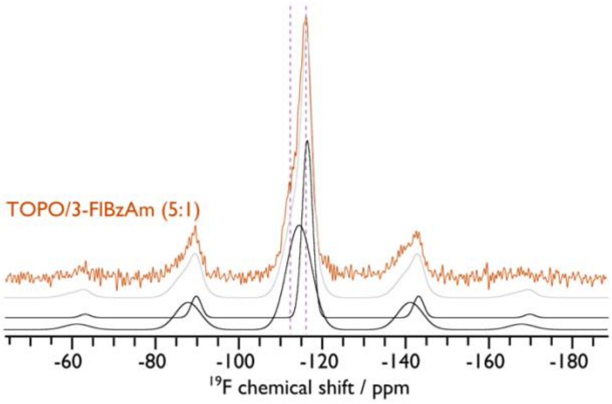

Figure S22. {^{1}H-}^{19}F CP MAS NMR spectrum of TOPO/3-FlBzAm (5:1) CdS NCs and associated fitting with deconvolution (black) and summation (grey). The positions indicated with the vertical dashed lines are the same as those given in Figure 5 of the main text.

Table S3. ^{19}F NMR parameters extracted from data fitting (see Figs. S15-S19). The values given for the span ($\Omega = \delta_{11} - \delta_{33}$) and skew ($\kappa = 3(\delta_{22} - \delta_{iso})/\Omega$) are not definitive owing to the large errors with the fitting.

**CdS nanocrystal system**	**^{19}F chemical shift (δ_{iso}) / ppm**	**^{19}F chemical shift span (Ω) / ppm**	**^{19}F chemical shift skew (κ)**	**Relative integral / %**
TOPO only	N/A	N/A	N/A	N/A
TOPO:3-FlAn (5:1)	–113.7	83	–0.2	100
TOPO:3-FlAn (1:1)	–113.7<br>–112.6	87<br>0	–0.2<br>N/A	97<br>3
TOPO:3-FlBzAm (64:1)	–116.0<br>–113.0	76<br>80	0<br>0	68<br>32
TOPO:3-FlBzAm (10:1)	–116.1<br>–112.9	64<br>76	0<br>0	81<br>19
TOPO:3-FlBzAm (5:1)	–117.5<br>–115.5	60<br>84	0<br>0	35<br>65

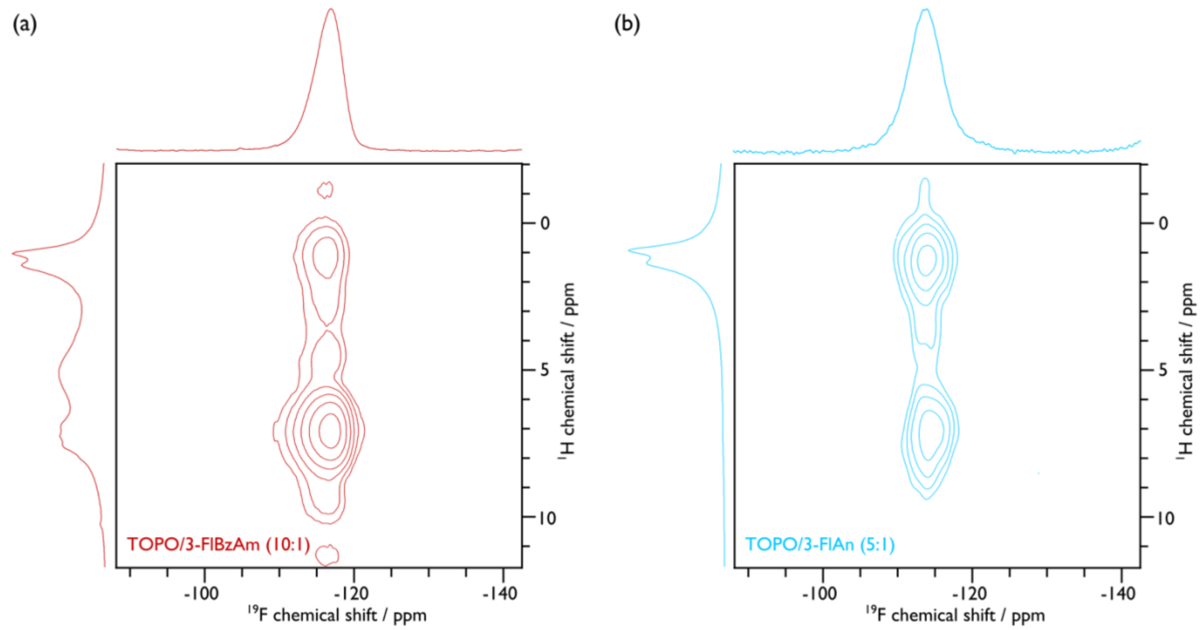

Figure S23. ^{1}H–^{19}F 2D dipolar correlation MAS NMR spectra of indicated CdS NCs. An MAS frequency of 12 kHz was used for both. Corresponding {^{1}H-}^{19}F CP (top) and ^{1}H (left) MAS NMR spectra are provided for comparison.

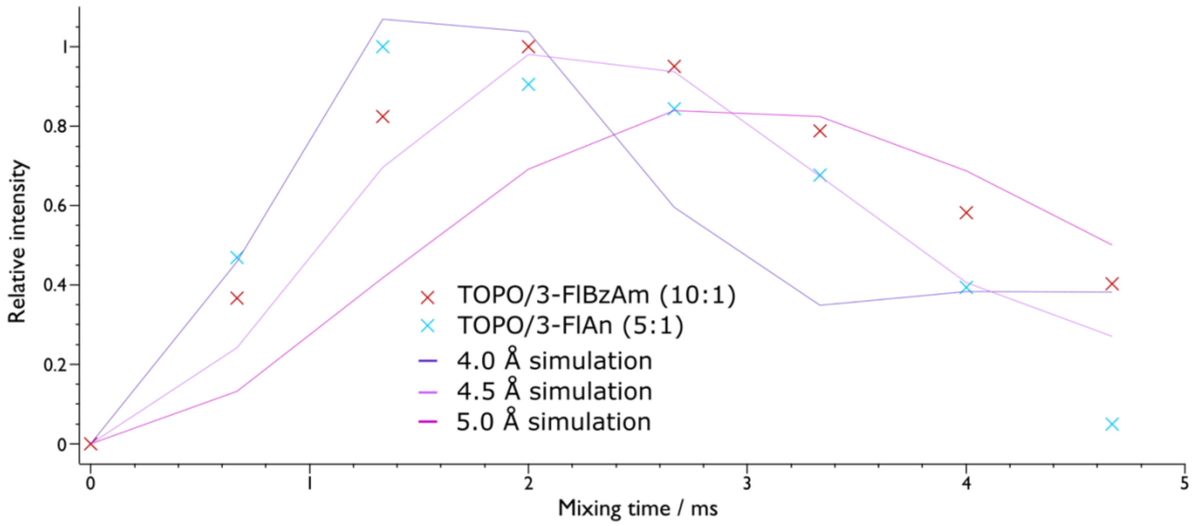

Figure S24. Plots of relative intensity of 1D ^{19}F homonuclear double-quantum-filtered dipolar correlation NMR spectra as a function of SPC-5[1] mixing time for (a) TOPO/3-FlBzAm (10:1) and (b) TOPO/3-FlAn (5:1) CdS NCs. An MAS frequency of 12 kHz was used for both datasets. Also shown are numerical simulations using SIMPSON[2] software for the corresponding intensity buildup curves for the indicated internuclear distances.

X-ray photoelectron spectroscopy

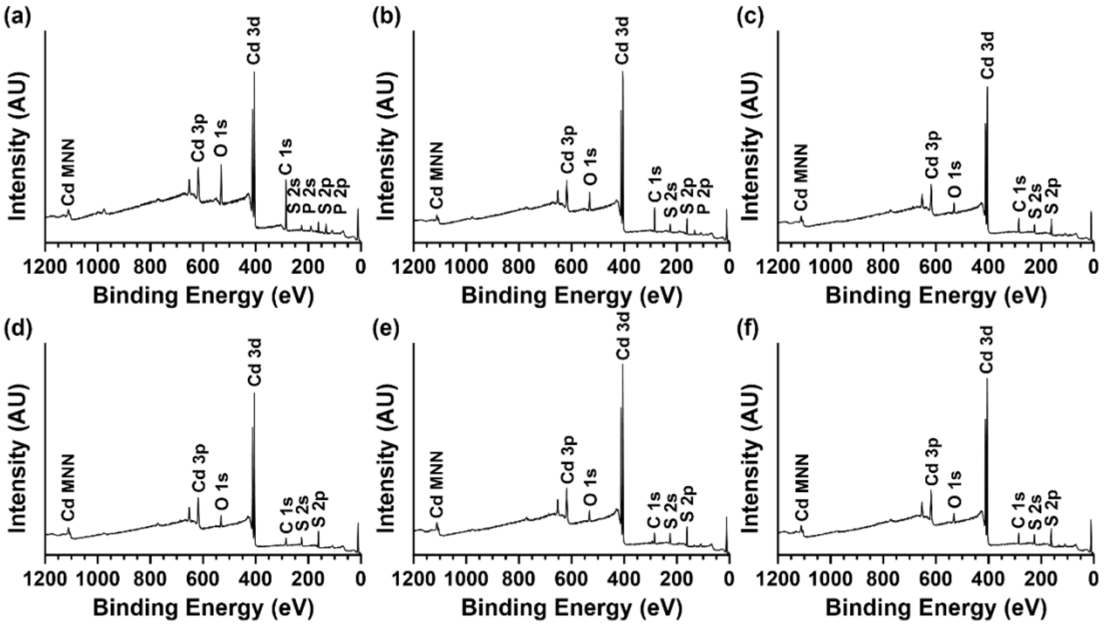

Figure S25. XPS survey spectra where (a) TOPO only, (b) 5:1 TOPO:3-FlAn, (c) 1:1 TOPO:3-FlAn, (d) 5:1 TOPO:3-FlBzAm, (e) 10:1 TOPO:3-FlBzAm and (f) 64:1 TOPO:3-FlBzAm are all shown.

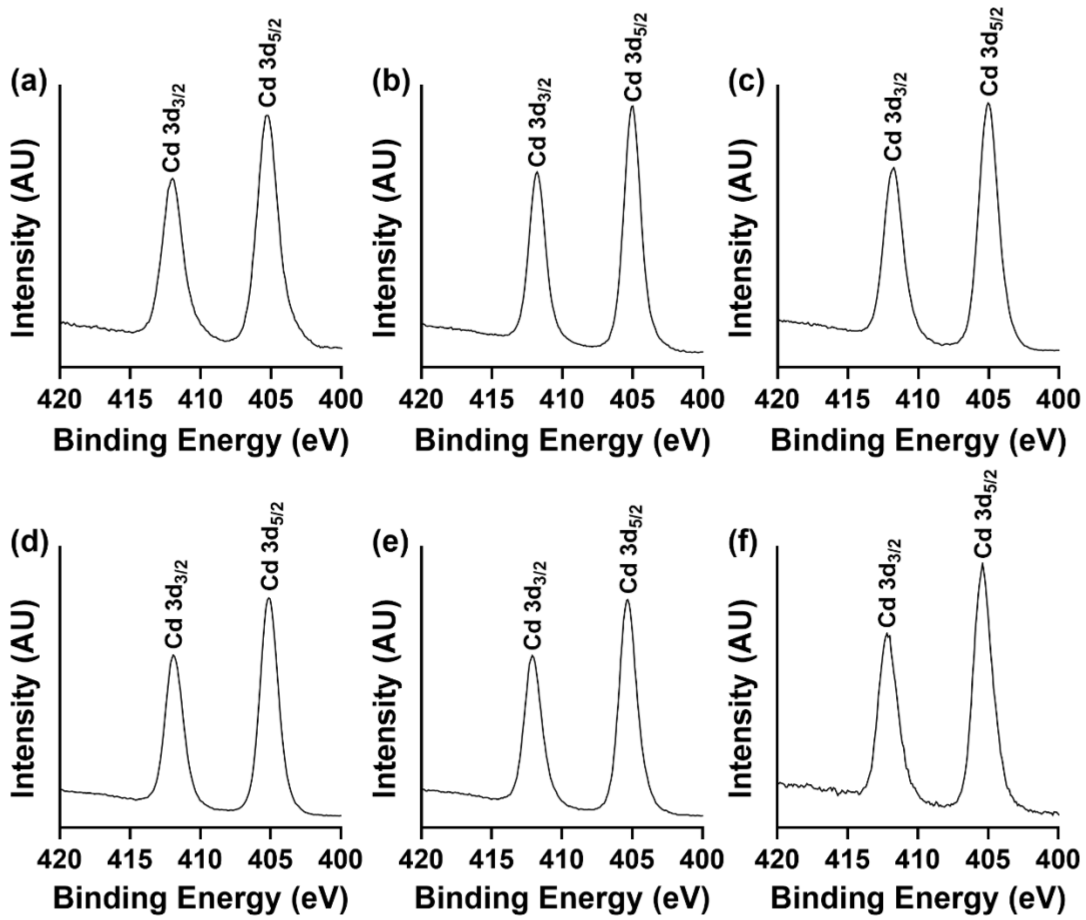

Figure S26. Cd3d of the XPS analysis where (a) TOPO only, (b) 5:1 TOPO:3-FlAn, (c) 1:1 TOPO:3-FlAn, (d) 5:1 TOPO:3-FlBzAm, (e) 10:1 TOPO:3-FlBzAm and (f) 64:1 TOPO:3-FlBzAm are all shown.

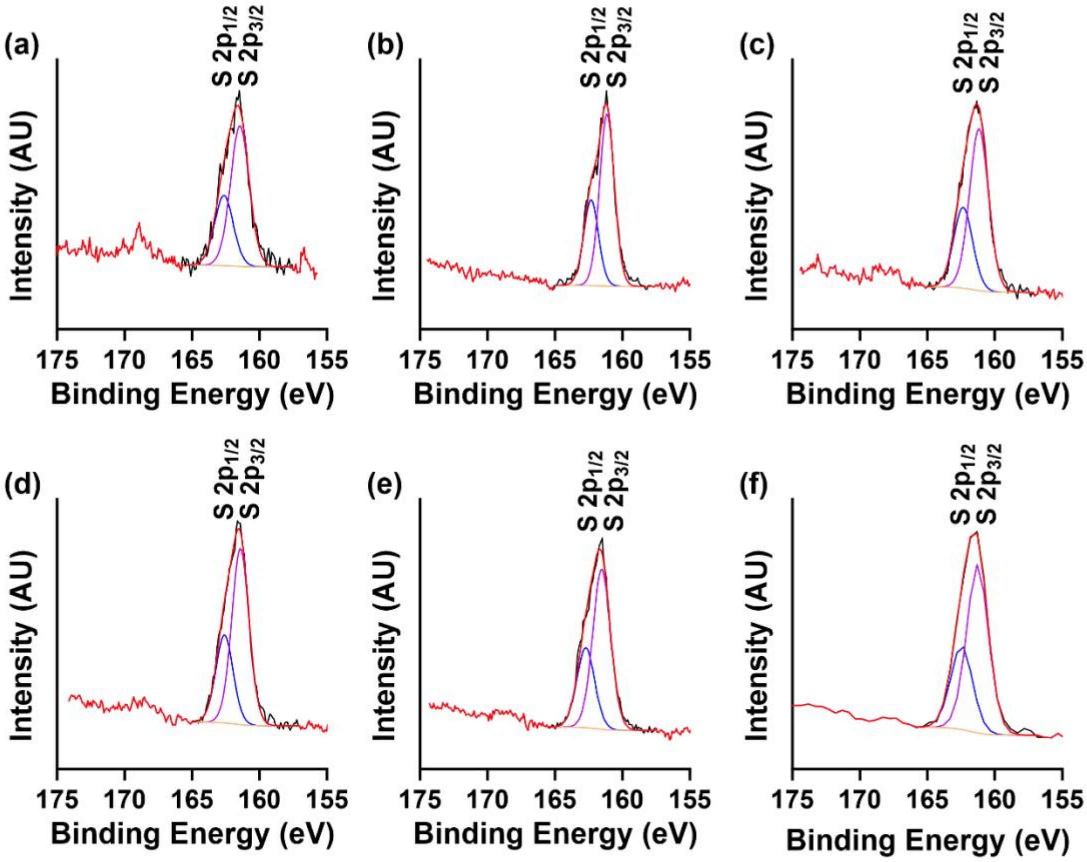

Figure S27. S2p of the XPS analysis where (a) TOPO only, (b) 5:1 TOPO:3-FlAn, (c) 1:1 TOPO:3-FlAn, (d) 5:1 TOPO:3-FlBzAm, (e) 10:1 TOPO:3-FlBzAm and (f) 64:1 TOPO:3-FlBzAm are all shown.

Table S4. Relative elemental amount from XPS quantification.

CdS NC system	C 1s %	Cd %	O 1s %	P 2p %	S 2p %
TOPO only	54.66	13.26	14.83	10.11	7.13
TOPO:3-FlAn (5:1)	40.02	21.20	12.66	7.77	18.35
TOPO:3-FlAn (1:1)	37.71	24.92	9.03	1.89	26.46
TOPO:3-FlBzAm (5:1)	25.62	30.98	12.06	0.93	30.41
TOPO:3-FlBzAm (10:1)	24.68	30.29	10.31	2.85	31.87
TOPO:3-FlBzAm (64:1)	27.14	28.47	10.41	4.37	29.61

Band gap analysis

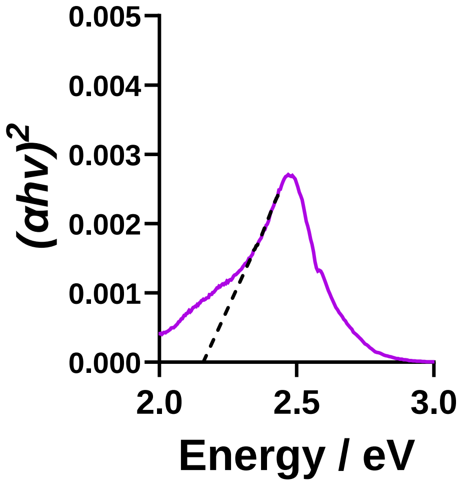

Figure S28. Calculated Tauc plot of the 64:1 TOPO:3-FlBzAm synthesised CdS nanocrystals suspended in toluene.

Table S5. Table of data for Scherrer analysis particle size and UV-Vis Tauc plot band gap energy of the synthesised CdS nanocrystals.

CdS nanocrystal system	Particle size (Scherrer) / nm	Band gap energy / eV
TOPO only	19	2.7
TOPO:3-FlAn (5:1)	37	2.2
TOPO:3-FlAn (1:1)	70	2.3, 3.7
TOPO:3-FlBzAm (64:1)	80	2.2, 4.2
TOPO:3-FlBzAm (10:1)	83	4.1
TOPO:3-FlBzAm (5:1)	200	4.0

References

1 M. Hohwy, C. M. Rienstra, C. P. Jaroniec and R. G. Griffin, *The Journal of Chemical Physics*, 1999, **110**, 7983–7992.

2 M. Bak, J. T. Rasmussen and N. Chr. Nielsen, *Journal of Magnetic Resonance*, 2011, **213**, 366–400.
